# miRNA expression profiles of premalignant and malignant arsenic-induced skin lesions

**DOI:** 10.1371/journal.pone.0202579

**Published:** 2018-08-16

**Authors:** Laila Al-Eryani, Samantha F. Jenkins, Vanessa A. States, Jianmin Pan, Janine C. Malone, Shesh N. Rai, Susan Galandiuk, Ashok K. Giri, J. Christopher States

**Affiliations:** 1 Department of Pharmacology and Toxicology, University of Louisville, Louisville, KY, United States of America; 2 Price Institute of Surgical Research, University of Louisville, Louisville, KY, United States of America; 3 Biostatistics Shared Facility, James Graham Brown Cancer Center, University of Louisville, Louisville, KY, United States of America; 4 Department of Medicine, University of Louisville, Louisville, KY, United States of America; 5 Department of Bioinformatics and Biostatistics, University of Louisville, Louisville, KY, United States of America; 6 Molecular Genetics Division, CSIR-Indian Institute of Chemical Biology, Kolkata, India; University of South Alabama Mitchell Cancer Institute, UNITED STATES

## Abstract

Arsenic, a naturally occurring element, contaminates the drinking water of over 200 million people globally. Chronic arsenic exposure causes multiple cancers including those originating from skin, lung and bladder, and is associated with liver, kidney, and prostate cancers. Skin is the primary target organ for arsenic toxicity; chronic toxicity initially manifests as non-malignant hyperkeratoses (HK) and subsequently advances to malignant lesions, including squamous cell carcinoma (SCC) and basal cell carcinoma (BCC). In this study, we evaluate the miRNA expression profiles of premalignant (3 HK) and malignant (3 BCC and 3 SCC) skin lesions from individuals chronically exposed to high levels of arsenic (59–172 ppb) in their drinking water in West Bengal, India. The lesions were histologically complex requiring histopathologic identification of keratinocytes to be isolated for RNA analyses. Keratinocytes were harvested using Laser Capture Microdissection and miRNA expression profiles were determined using TaqMan® Array Human MiRNA A Card v2.0. Thirty-five miRNAs were differentially expressed among the three lesion types analyzed. Two miRNAs (miR-425-5p and miR-433) were induced in both BCC and SCC relative to HK indicating their association with malignancy. Two other miRNAs (miR-184 and miR-576-3p) were induced in SCC relative to both BCC and HK suggesting selective induction in tumors capable of metastasis. Six miRNAs (miR-29c, miR-381, miR-452, miR-487b, miR-494 and miR-590-5p) were selectively suppressed in BCC relative to both SCC and HK. In conclusion, the differential miRNA expression was both phenotype- and stage-related. These miRNAs are potential biomarkers and may serve as therapy targets for arsenic-induced internal tumors.

## Introduction

Arsenic is a naturally occurring element that is prevalent in the earth’s crust [1]. Exposure to arsenic in drinking water is a worldwide problem; more than 200 million people consume water contaminated with arsenic above the World Health Organization (WHO) recommended limit (10 μg/L, in 2008) [2]. According to the WHO, exposure to levels of arsenic exceeding the safe limit for more than six months leads to arsenicosis. Geographic areas with high levels of arsenic in drinking water are mainly in South Asia (Bangladesh, India, Nepal, Cambodia, Viet Nam, Taiwan) and Latin America (Argentina, Bolivia, Chile, Mexico) and to a lesser extent, the U.S.A. [3–7]. The Ganges River delta spanning West Bengal in India and Bangladesh is the region associated with the largest mass poisoning in human history from arsenic contaminated drinking water coming from natural sources [8]. More than 70 million people in the delta area consume highly arsenic contaminated drinking water and have high-risk of arsenicosis and a wide range of chronic arsenic exposure-associated health complications such as pregnancy complications and teratogenicity, developmental effects, neurotoxicity, diabetes, pulmonary disease, cardiovascular disease, skin manifestations and cancer [8, 9]. Arsenic has been classified for more than 15 years by the International Agency for Research on Cancer (IARC) as a Group 1 human carcinogen [10] and Group A by the EPA [11]. Chronic arsenic exposure causes multiple cancers including those originating from skin, bladder and lung, and to a lesser extent, liver, kidney, and prostate [10].

Skin cancer is the most prevalent form of all cancers [12]. Most skin cancers are a consequence of mutations resulting from DNA damage caused by the ultraviolet radiation in sunlight [13]. The second most frequent cause of skin cancer is chronic arsenic exposure. In populations exposed to high levels of arsenic in their drinking water and food, arsenic is a leading cause of skin cancer [3]. The skin is the primary target organ for arsenic toxicity and Sir Jonathan Hutchinson was the first to report skin cancer in patients consuming arsenic-based medications in 1887/88 [14]. Arsenic-induced skin toxicity symptoms first appear as pigmentary changes including raindrop-shaped lesions and diffuse dark brown lesions followed by arsenical keratosis involving the palms, soles and trunk, and multiple cutaneous malignancies [15]. Non-malignant arsenic-induced diseases are also described, including Blackfoot disease, a dry gangrene resulting from ischemic changes in the toes accompanied by ulcers [16].

**Fig 1:**
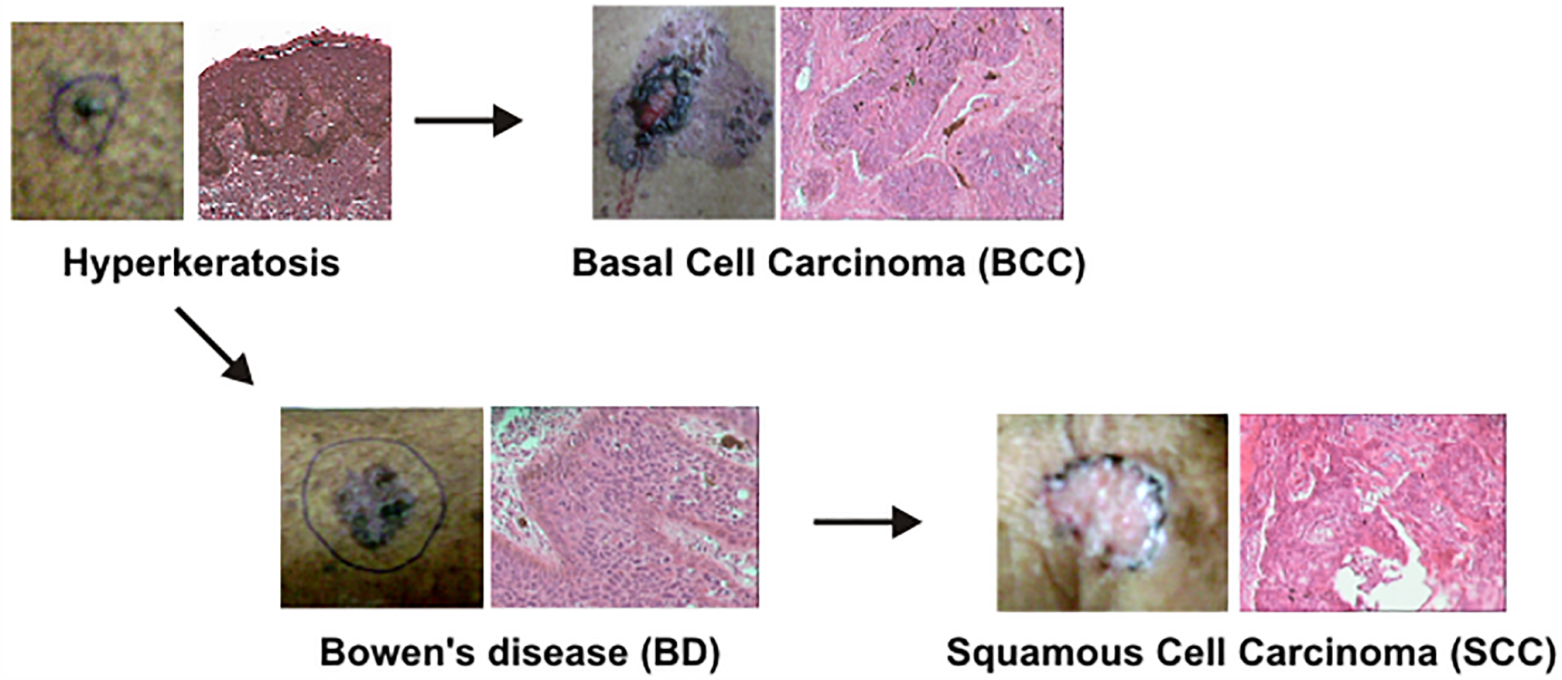
Arsenic-induced skin cancer progression. Progression from the premalignant hyperkeratosis lesion (HK) to either of the malignant lesions, basal cell carcinoma (BCC) directly or squamous cell carcinoma (SCC) through Bowen’s disease (BD, SCC *in situ*). Shown are photographs of lesions *in situ* prior to excision with a histological section of the formalin fixed and paraffin embedded sample stained with hematoxylin and eosin on the right.

Arsenic-induced skin cancer has a different pattern of pathology and progression to malignancy compared to sunlight-induced skin cancer. Actinic keratoses are the premalignant lesions for sunlight-induced squamous cell carcinoma (SCC) while arsenical hyperkeratoses are premalignant lesions of both basal cell carcinoma (BCC) and SCC in arsenicosis (Fig 1) [13, 17]. Moreover, sunlight causes SCC, BCC and malignant melanoma whereas arsenic exposure is not associated with malignant melanoma. A keratosis or hyperkeratosis in body areas not exposed to the sun is the most common cutaneous manifestation of arsenicosis [15]. Pathologically, arsenical keratoses are characterized by pathological features such as hyperkeratosis, parakeratosis, arsenical pigmentation, and SCC *in situ* at a later stage [18]. The color of keratoses is usually the normal skin color or darker, the size of lesions is dependent on the disease progression stage, and larger lesions generally are more severe [15]. The presence of dysplasia in the keratotic lesions is an intermediate stage between premalignancy and malignancy and histological characterization can be challenging. Dysplastic keratoses are immediate predecessors to Bowen’s disease (BD) and SCC. Chronic arsenic exposure also causes BD, which is analogous to SCC *in situ* [15]. BD and superficial BCC are the most common malignant lesions in arsenicosis [15]. SCC may progress to invasion in later stages of arsenic exposure [15, 18]. Individuals can have one or several types of skin lesions simultaneously [15].

The mechanisms of arsenic-induced carcinogenesis are not yet clear and there are several proposed mechanisms including epigenetic alterations. MicroRNAs (miRNAs) are part of the epigenome and play a fundamental role in the regulation of most mammalian protein codling genes [19]. miRNAs are a family of small noncoding RNAs between 21 and 25 nucleotides in length. Multiple studies on several cancers such as breast, ovary, lung, bladder and colon cancers showed that many miRNAs play an important role in carcinogenesis serving as oncogenes and tumor suppresser genes as well as serving as markers for disease diagnosis [20]. To add to the complexity of miRNAs’ role in regulating gene expression, one miRNA can target several mRNAs and an individual mRNA can be targeted by multiple miRNAs creating complex regulatory loops in a cell establishing a balance across gene networks [21]. Furthermore, the association between miRNA expression and tumor development, progression and response to therapy has been the subject of several studies supporting miRNAs as potential biomarkers for disease diagnosis and prognosis [20]. The current study demonstrates an association between miRNA expression and arsenic-induced skin lesion development and progression, and supports miRNA expression as a marker for the development and progression of stages of the carcinogenic process.

## Materials and methods

### Sample collection criteria and diagnosis

Skin lesion samples were obtained as 2 mm skin punch biopsies from tumors and lesions excised for diagnostic purposes from subjects visiting field hospitals in the Murshidibad district of West Bengal, India. Samples were collected at various times from 2008 to 2009 and fixed samples were stored as paraffin blocks. Criteria for inclusion in this study were that patients were from villages with arsenic contaminated drinking water and exhibited skin symptoms of chronic arsenicosis. Samples were collected with informed consent provided in native language with approval of the Ethical Committee on Human Subjects at Indian Institute of Chemical Biology and the Institutional Review Board at University of Louisville. The samples were fixed in formalin (light fixation) and embedded in paraffin (FFPE). Clinical diagnosis of the samples was performed by dermatologists in India at the time of lesion excision, and the histopathologic diagnosis was confirmed by a dermatopathologist in Louisville examining thin sections stained with hematoxylin and eosin (H&E). All samples analyzed for miRNA expression were superficial lesions. Staging was determined based on histopathology.

### Laser capture microdissection

RNA purification from samples was conducted at various times from 2014 to 2017. Samples were 5 to 8 years old at the time of RNA purification from thin sections cut from paraffin blocks. Keratinocytes were isolated from 7 μm sections of samples using an ArcturusXT™ Laser Capture Microdissection System. The sectioned tissues were first deparaffinized and stained using the Arcturus Paradise Plus staining kit and the isolated cells were collected on macrocaps. Total RNA was purified from the tissues on the macrocaps using Arcturus Paradise Plus FFPE RNA Extraction and Isolation Kits. Total RNA quality and quantity were determined using the Agilent RNA 6000 Pico Kit, Eukaryote, version 2.6 and the Agilent 2100 Bioanalyzer instrument (Agilent Technologies, Inc., Santa Clara, CA, USA). Total RNA (50–100 ng) from each sample was reverse transcribed and pre-amplified to prepare cDNA using RT kit, MegaPlex Primers and Pre-Amp kit (Life Technologies) following the Megaplex™ Pools For microRNA Expression Analysis protocol with pre-amplification.

### miRNA RT-qPCR array cards

Profiling miRNA expression was performed using the TaqMan® Array Human MiRNA A Card v2.0 (polymerase chain reaction (RT-qPCR) array cards, Life Technologies). The array measures the expression of 384 targets; 377 miRNAs and 7 controls. Four of seven controls are U6 RNA, the mean of which was used for normalization in our dataset. The data were collected at 0.1 threshold value on an Applied Biosystems ViiA7 Real Time PCR System. The Ct values were normalized to the mean of Ct values of U6 RNA (ΔCT method). The data have been deposited in the National Center for Biotechnology Information Gene Expression Omnibus (NCBI-GEO) under accession number GSE102892.

### Statistical analysis

The ΔCt values were calculated using the Ct values of the miRNAs from premalignant or malignant lesions and normalizing them to the means of the Ct values of the reference RNA U6. Statistical analyses were performed by comparing the ΔCt values of the premalignant and malignant lesions using one-way ANOVA by SAS System V9. Cary, NC: SAS Institute Inc, 2003.

## Results

### Study population demographics and histopathological analysis

The subjects were exposed to high levels of arsenic in their drinking water (51 to 398 ppb; mean 106.4 ± 63.9), which is similar to well water arsenic levels seen in the northeastern and southwestern United States [22–24]. Skin lesion samples were collected from 35 males (25–60 years old) with urine arsenic levels ranging from 100 to 1,590 μg/L (mean 299.3 ± 281.9). The subset from which miRNAs in lesions were analyzed had narrower ranges of age, water and urine arsenic (47 ± 3.9, 92.7 ± 41.3, 335.8 ± 324.9 respectively, Table 1). Diagnostic and demographic information for these samples also are listed in Table 1.

**Table 1:** Demographic and diagnostic data on arsenic-induced skin lesion samples used for miRNA analyses.

Sample ID	Diagnosis	Stage	Gender	Age	Water Arsenic (ppb)	Urine Arsenic (μg/L)
14E	HK	benign	M	50	91.1	240
28E*	HK	benign	M	40	61.5	140
19D^#^	HK	benign	M	50	59.8	417
28B*	SCC	TisN0M0	M	40	61.5	140
26A^#^	SCC	T1N0M0	M	50	59.8	417
18B^$^	SCC	TisN0M0	M	45	79.8	100
8A^$^	BCC	superficial	M	45	79.8	100
17A	BCC	superficial	M	49	172	958
20C	BCC	superficial	M	48	92	160

HK = benign hyperkeratosis, SCC = squamous cell carcinoma, BCC = basal cell carcinoma

^*, #^ and ^$^ indicate paired samples from the same individual.

**Fig 2:**
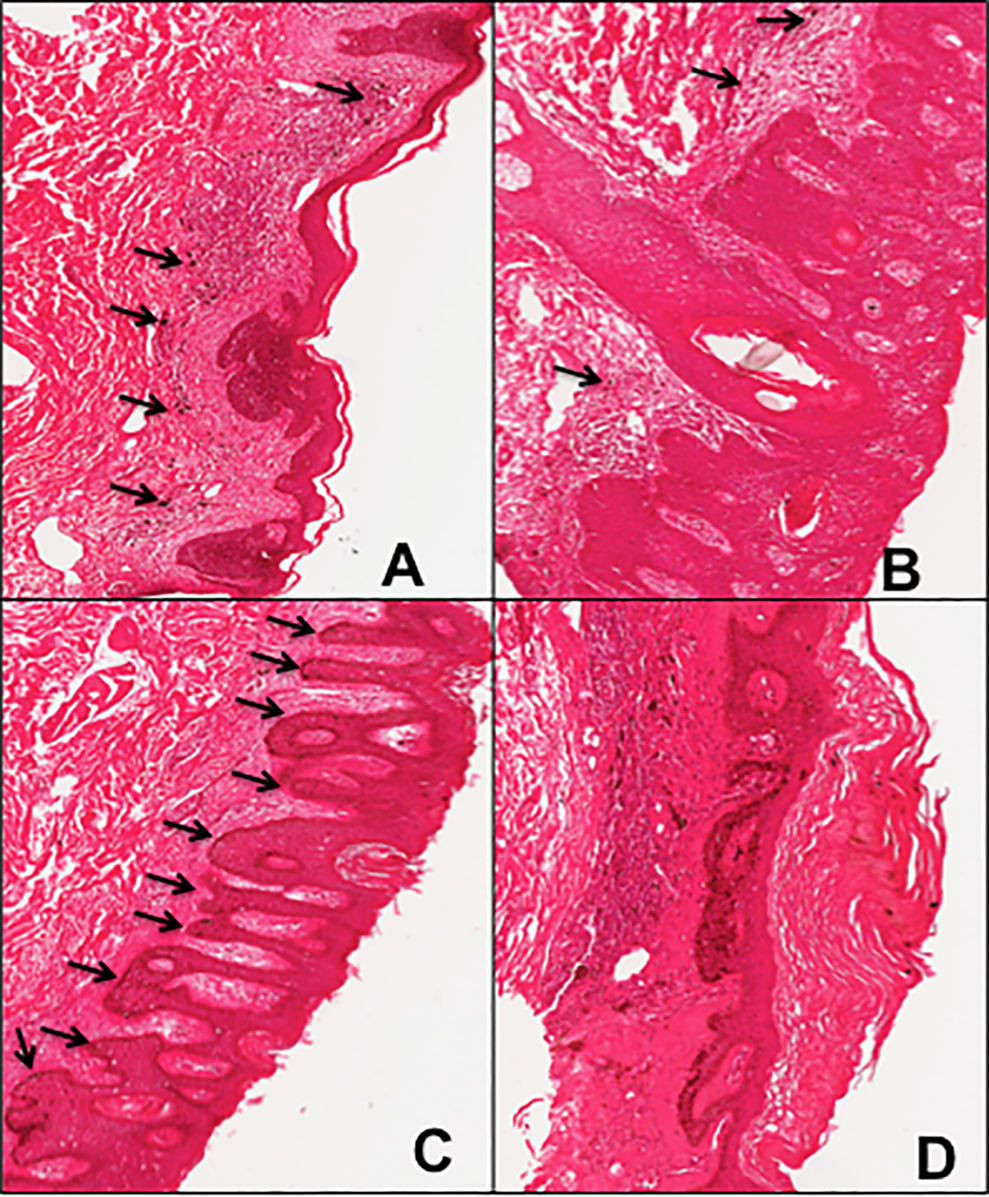
Arsenic induced premalignant and malignant skin lesions. Histological sections of arsenic-induced basal cell carcinoma, squamous cell carcinoma and hyperkeratosis. **A.** Basal cell carcinoma, arrows point to inflammatory foci. **B.** Squamous cell carcinoma, arrows point to inflammatory foci. **C.** Hyperkeratosis with dysplasia, arrows point to areas of dysplasia projecting from the hyperkeratotic region. **D.** Hyperkeratosis without dysplasia.

**Fig 3:**
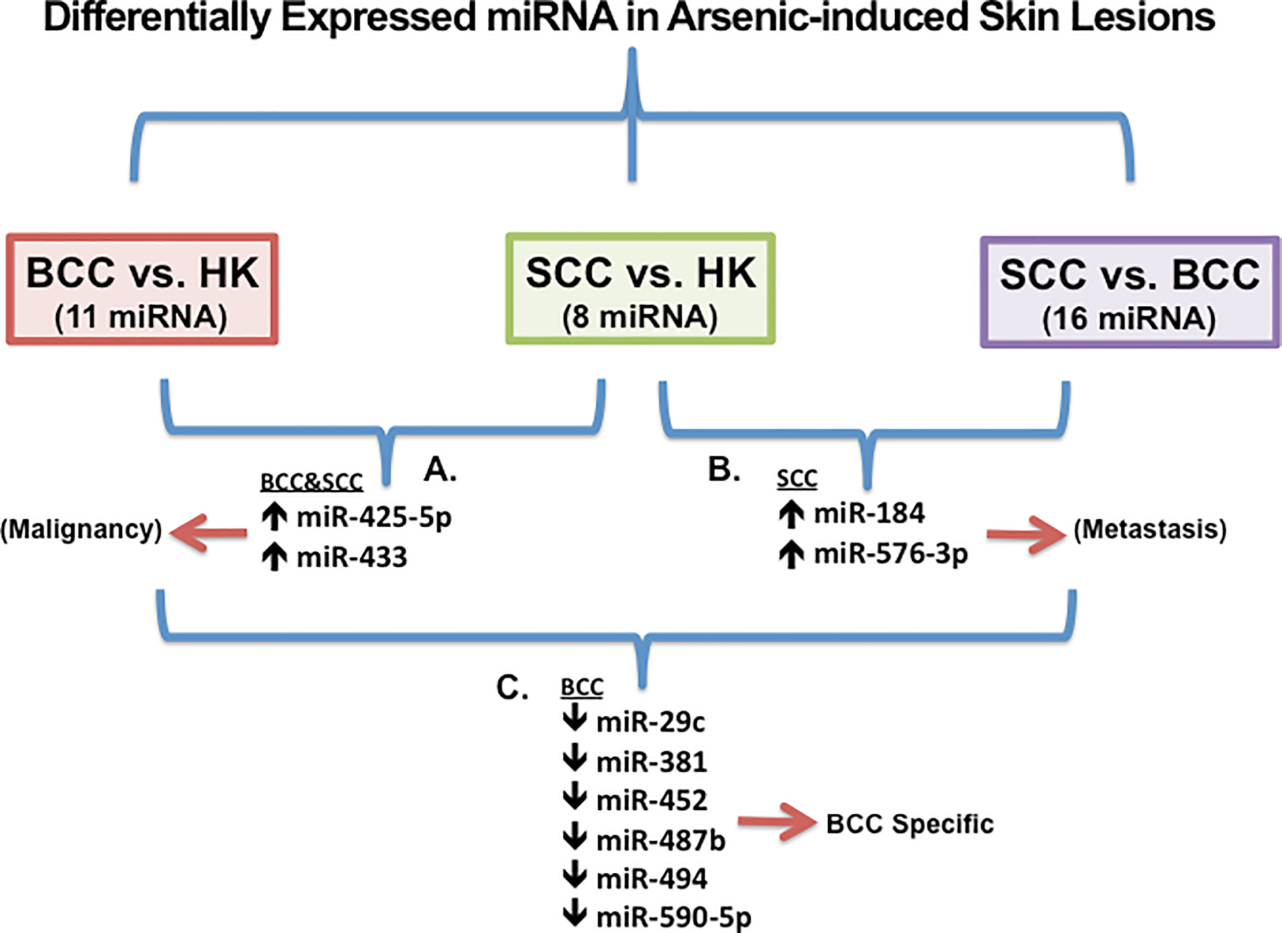
Differentially expressed miRNA in arsenic-induced skin lesions. Differential expression comparisons are diagrammed. Differentially expressed miRNAs in: **A**. malignant lesions, both BCC and SCC relative to HK; **B**. Specific to SCC vs HK or BCC; **C.** Specific to BCC vs SCC or HK. Direction of expression changes indicated by arrows. Additional differentially expressed miRNAs are listed in S1 Table.

The skin lesions were histologically complex, often containing variable obscuring inflammatory infiltrates, thus requiring histopathologic identification of keratinocytes to be isolated for RNA analyses. Inflammatory infiltrates were commonly observed in BCC (Fig 2A) and SCC (Fig 2B) samples. Many of the lesions classified as hyperkeratosis on clinical examination contained regions of dysplasia bordering on early Bowen’s disease (Fig 2C). The HK samples were divided into 2 groups based on histology. The first showed dysplasia and were excluded from the selection (Fig 2C). The second were samples that showed no signs of dysplasia (true premalignant lesions, Fig 2B). HK that showed no signs of dysplasia (true premalignant lesions) were selected for further processing. Clinical diagnoses (BCC, SCC and BD) were confirmed histologically. Keratinocytes were harvested from each sample group using Laser Capture Microdissection (LCM). LCM technology permitted microscopic precision and the ability to harvest exclusively pathology-proven diseased tissue from biopsy samples while excluding dysplastic cells and inflammatory and other non-keratinocyte tissues (Fig 3). Most samples yielded too little RNA for analysis. Three samples of each lesion type (HK, BCC, SCC) yielded adequate amounts of RNA for analysis.

### miRNA profiles in premalignant and malignant HK, BCC and SCC lesions

Differential miRNA expression profiles were obtained by comparing expression in three premalignant HK lesions and six malignant lesions (3 SCC and 3 BCC). One-way ANOVA analysis of ΔCt values (vs U6 RNA) indicated that thirty-five miRNAs were differentially expressed among the three lesion types analyzed (Fig 3, S1 Table). Differential expression fell into six classifications (S1 Table). Two miRNAs were induced in both BCC and SCC relative to HK (Fig 3A, Table 1), e.g. these miRNAs were associated with malignancy in general. Two other miRNAs were induced in SCC relative to both BCC and HK (Fig 3B, Table 1), e.g. these were selectively induced in the tumors capable of metastasis. Six miRNAs were selectively suppressed in BCC relative to both SCC and HK (Fig 3C, Table 1). A fourth group of three miRNAs were differentially expressed in BCC relative to HK, but expression in SCC was not different from either BCC or HK (S1 Table). A fifth group of three miRNAs were differentially expressed in SCC relative to HK, but expression in BCC was not different from either SCC or HK (S1 Table). The sixth group contained eight miRNAs induced in SCC relative to BCC, but not differentially expressed in HK relative to either SCC or BCC.

## Discussion

Ultraviolet light in sunlight is the most common cause of skin cancer. Exposures have a cumulative mutagenic effect on cellular DNA. Carcinogenesis has been demonstrated when cellular function is disturbed by mutations in tumor suppressors, oncogenes, transcription factors, and nucleotide excision repair proteins [25]. The mechanism(s) of arsenic-induced skin cancers is not completely characterized, but evidence suggests that mutations are not the main driving force [26, 27]. Other pathways have been hypothesized including DNA repair inhibition, cell cycle pathway dysregulation and epigenetic modification including histone acetylation, methylation, phosphorylation and differential miRNA expression [28–31]. In particular, dysregulation of miRNA expression has been implicated in a wide variety of cancers [20].

In the current study, we sought to investigate the potential role of dysregulation of miRNA expression in arsenic-induced carcinogenesis. Differential miRNA expression was characterized in human arsenic-induced skin lesion samples collected from individuals exposed to high levels of arsenic in their drinking water. An important observation is that the histopathology of the samples revealed potential confounding characteristics of the lesions especially for the premalignant arsenic-induced skin lesions. The histological diagnosis of the HK samples was very critical because many of our HK samples showed dysplasia with some even resembling early Bowen’s disease. These samples were excluded from the miRNA analyses. Three HK lesions with no signs of dysplasia were selected for further processing and their profiles were compared to malignant BCC and SCC lesions. The age of the samples resulted in loss of RNA integrity reducing yield. Many samples yielded insufficient amounts for the RT-qPCR array analysis. Thus, a weakness of the current study is the small number of samples analyzed. In spite of this limitation, we were able to obtain miRNA expression profiles distinguishing the malignant BCC and SCC from the premalignant HK.

The results showed differential expression of 35 miRNAs in the premalignant and malignant lesions. Some of differentially expressed miRNAs were lesion or stage specific. For example, expression of miRNAs miR-425-5p and miR-433 was higher in both BCC and SCC relative to HK, with no significant difference in expression between SCC and BCC. This result suggests that increased expression of miR-425-5p and miR-433 is associated with progression from premalignant to malignant lesions (Fig 3A, S1 Table). Consistent with this suggestion, miR-425-5p is an intronic miRNA embedded within the *DALRD3* (DALR anticodon binding domain containing 3 gene) and has been reported to be induced in metastatic gastrointestinal cancers such as gastric and colorectal cancer and possibly associated with melanoma [32–34]. miR-433 is embedded antisense within *RTL1* (retrotransposon Gag like 1) and is associated with poor progression-free survival in high-grade serous ovarian cancer patients [35]. miR-433 is also induced in metastatic bladder cancer [36] suggesting it is acting as an oncogene, but it is suppressed in gastric cancer suggesting it is functioning as a tumor suppressor gene in the pathogenesis of this cancer type [37].

Similarly, two miRNAs, miR-184 and miR-576-3p, were induced in SCC relative to BCC and HK lesions suggesting that increased expression is associated with the invasive and potentially metastatic phenotype of SCC (Fig 3B, S1 Table). miR-184 is embedded antisense to *ANKRD34C-AS* (ANKRD34C antisense RNA 1) and was reported to be induced in several squamous cell carcinomas including SCC involving the tongue [38] and head and neck [39], as well as in glioma [40], and hepatocellular carcinoma [41, 42]. miR-184 was reported to be suppressed in prostate carcinoma [43], neuroblastoma [44], epithelial ovarian cancer [45], and renal cell carcinoma [46]. Thus, the role of this miRNA as oncogene or tumor suppressor may be context specific. miR-576-3p is an intronic miRNA embedded within *SEC24B* (SEC24 homolog B, COPII coat complex component gene) and is suppressed in bladder cancer [47], T-cell precursor acute lymphoblastic leukemia [48], and sera of non-melanoma skin cancer patients (BCC and SCC, UV light related) [49].

Six miRNAs (miR-29c, miR-381, miR-452, miR-487b, miR-494 and miR-590-5p) were suppressed in BCC relative to both SCC and HK lesions suggesting that their suppression is specific to BCC phenotype (Fig 3C, S1 Table). miR-29c is embedded within last exon of *C1orf132* (chromosome 1 open reading frame 132), and the transcript encodes an unknown open reading frame. miR-29c was found induced in serum of non-small cell lung cancer patients (S1 Table). However, consistent with its suppression in BCC, miR-29c was reported to be suppressed in several cancers including bladder cancer, esophageal SCC, gastric cancer, head and neck SCC, hepatocellular carcinoma, lung adenocarcinoma, nasopharyngeal carcinoma, and glioma (S1 Table).

miR-381 and miR-487b were both suppressed in BCC. These miRNAs, along with four other miRNAs (miR-539, miR-889, miR-544a, miR-655), are part of the MIR381 host gene (MIR381HG). These other four miRNAs are all 3’ of miR-381 and miR-487b and were not differentially expressed among our HK, BCC and SCC samples. Although miR-381 has been reported to be induced in glioma, this miRNA is reported as suppressed in other tumors including oral SCC, epithelial ovarian cancer, hepatocellular carcinoma, colorectal cancer, gastric cancer, breast cancer, colon cancer and lung adenocarcinoma (S1 Table) consistent with its decreased expression in BCC in our study. Likewise, we saw that miR-487b-3p was suppressed in BCC and it has been reported as suppressed in colon cancer, pediatric glioma and metastatic prostate cancer (S1 Table). miR-452-5p is embedded in an intron of *GABRE* (gamma-aminobutyric acid type A receptor epsilon subunit gene) and was suppressed in BCC. As for several other miRNAs suppressed in BCC, miR-452-5p was induced in several malignancies (hepatocellular carcinoma, clear cell renal cell carcinoma and bladder cancer, but found to be suppressed in several other cancers and sarcomas (lung adenocarcinoma, chondrosarcoma, osteosarcoma, and prostate cancer (S1 Table). miR-494-3p is one of a cluster of five miRNAs on chromosome 14, but only miR-494 was found to be differentially expressed in HK, BCC or SCC. Consistent with this finding, miR-494 was reported as suppressed in malignant breast cancer, gastric carcinoma, ovarian cancer, and pancreatic cancer (S1 Table). Contrary to our finding of miR-494 being suppressed in BCC relative to SCC and HK, miR-494 is induced in cervical cancer and colorectal cancer (S1 Table). Once again, the relationship of miR-494 expression to oncogenesis appears to be context specific. Furthermore, rather than inferring its suppression in BCC, perhaps miR-494 is induced early in the transformation of keratinocytes to SCC.

miR-590-5p is embedded in *EIF4H* (eukaryotic translation initiation factor 4H gene). Like several other miRNAs we found suppressed in BCC, miR-590-5p was found to be induced in several cancers (cervical cancer {, gastric cancer, vulvar squamous cell carcinoma, renal cell carcinoma (S1 Table)) and suppressed in others (malignant melanoma, breast cancer, colorectal cancer (S1 Table)). Thus, the weight of evidence suggests that these miRNAs suppressed in BCC likely are tumor suppressors. This inference is supported by the observations of others that these miRNAs are suppressed in a wide variety of other tumor types.

The three miRNAs with the greatest change in expression in SCC relative to HK and BCC were miR-520f and miR-184 (induced) and miR-127-5p (suppressed) with fold changes of 7973, 1373 and 207 965, respectively (S1 Table). miR-520f is an intergenic miRNA located on Chromosome 19. miR-520f was found to be induced in glioblastoma tumor cells and suppressed in gastric carcinoma (S1 Table). In our data set, miR-520f was induced in SCC relative to BCC lesions suggesting its possible association with invasive properties of SCC. However, a study on PANC-1 cells (human pancreatic carcinoma cell line) showed that overexpression of miR-520f led to reversal of epithelial-to-mesenchymal transition, exerting anti-invasive and antimetastatic effects {van Kampen, 2017 #205}. Thus, the potential role of miR-520f in arsenic-induced SCC is unclear. A study by Wong et al. on SCC cell lines originating in tongue suggested that miR-184 is an oncogenic miRNA or an “oncomir” [38]. The study showed that the inhibition of miR-184 leads to reduction of cell proliferation and increased apoptosis in three SCC cell lines [38]. miR-184 was induced in our SCC samples compared to both HK and BCC samples suggesting that it is an oncomir that may play a role in cell invasion considering the invasive nature of SCC tumors.

miR-127-5p was found suppressed in breast cancer, gastric cancer, hepatocellular carcinoma and colon cancer patients’ stool (S1 Table). These results suggest that miR-127-5p is a tumor suppressor. miR-127-5p was suppressed only in our SCC samples suggesting that it may be more relevant to the invasive properties of SCC. This suggestion is supported by a study showing that miR-127-5p suppresses MMP-13 and IL-1β responses in human chondrocytes and the decreased expression of miR-127-5p enhances the progression of cartilage destruction [50].

Not only does arsenic-driven skin cancer have a different pattern of pathology and progression to malignancy than sunlight-induced skin cancer, but also different miRNA (miRNA) expression profiles. The miRNA expression of miR-576-3p reported by Balci et al. for sunlight-induced BCC and SCC is not reflected in the miRNA expression profiles of arsenic-induced BCC and SCC in our study [49], providing further support for the difference in etiology between arsenic-induced and sunlight-induced skin cancers.

In summary, this study is the first to characterize and identify miRNAs in human arsenic-induced pre-malignant and malignant skin lesions. The results provide original information profiling changes in miRNA expression associated with arsenic-induced transformation from premalignant HK to malignant SCC and BCC. The results show that the expression of some miRNAs was phenotype related, e.g. SCC or BCC, or stage related, e.g. pre-malignant (HK) or malignant (SCC). Our results provide insight on the potential role of miRNAs in arsenic-induced carcinogenesis in general and in arsenic-induced skin cancer in particular. Furthermore, the results suggest that some of the miRNAs we described could be potential biomarkers or therapy targets for arsenic-induced internal cancers. Therefore, further investigation is required to understand their mechanistic role in the carcinogenesis process resulting from chronic arsenic exposure.

## Supporting information

S1 Table Differentially expressed miRNA in Arsenic-induced skin lesions and their expression reported in other types of human cancers. (PDF)
