# Supplementary material for: miRNA expression profiles of premalignant and malignant arsenic-induced skin lesions
Source: PLoS One. 2018 Aug 16;13(8):e0202579. doi: 10.1371/journal.pone.0202579 (PMC6095593; doi:10.1371/journal.pone.0202579)
Supplement: S1 Table — (PDF) [file pone.0202579.s001.pdf]

Supplementary Table 1: Differentially expressed miRNAs in Arsenic-induced Skin Lesions and Their Expression Reported in Other Types of Human Cancers.

miRNA	Type of miRNA (location)	Direction and fold change of expression	Type of cancer and direction of expression change in other studies	References
**Differential expression BCC and SCC vs. HK (malignant lesions associated miRNAs)**				
miR-425-5p	Intronic (embedded within DALRD3)	↑31 (BCC vs. HK)<br><br>↑56 (SCC vs. HK)	↑Gastric cancer, colorectal cancer<br>Suggested biomarker in melanoma	[1-3]
miR-433	Antisense within RTL1 retrotransposon Gag like 1	↑152 (BCC vs. HK)<br><br>↑26 (SCC vs. HK)	Associated with poor progression-free survival in High-grade serous ovarian cancer (deregulated in ovarian cancer)<br>Tumor suppressor role in liver cancer<br>↓Gastric cancer<br><br>↑Bladder cancer	[4-8]
Differential expression SCC vs. BCC and HK (invasion associated miRNAs)				
miR-184	Antisense to ANKRD34C antisense RNA 1	↑1373 (SCC vs. BCC)<br><br>↑368 (SCC vs. HK)	↑Tongue SCC and glioma, invasive breast cancer, in sebaceous carcinomas compared with sebaceous adenomas, hepatocellular carcinoma, head and neck squamous cell carcinoma<br><br>↓Prostate carcinoma, breast cancer, neuroblastoma, epithelial ovarian cancer, non-small cell lung cancer and small cell lung cancer, Renal cell carcinoma	[9-22]

miR-576-3p	Intronic (embedded within SEC24B)	↑31 (SCC vs. BCC)<br><br>↑55 (SCC vs. HK)	↓Bladder cancer, sera of nonmelanoma skin cancer patients (BCC and SCC, UV light related), T-cell precursor acute lymphoblastic leukemia	[23-25]
Differential expression BCC vs. HK and SCC (selectively suppressed in BCC)				
miR-29c-3p	Within last exon of C1orf132, chromosome 1 open reading frame 132 along with miR-29B2	↓111 (BCC vs. HK)<br><br>↓109 (BCC vs. SCC)	↑Non-small cell lung cancer<br><br>↓Bladder cancer, esophageal squamous cell carcinoma, Gastric cancer, glioma, head and neck squamous cell carcinoma, hepatocellular carcinoma, Lung adenocarcinoma, nasopharyngeal carcinoma, high-metastatic lung cancer, pancreatic cancer	[26-36]
miR-381-3p	Part of MIR381 host gene (MIR381HG)	↓241 (BCC vs. HK)<br><br>↓181 (BCC vs. SCC)	↑Osteosarcoma, glioma<br><br>↓Oral squamous cell carcinoma, Epithelial ovarian cancer, hepatocellular carcinoma, colorectal cancer, gastric cancer, breast cancer, renal cell cancer, colon cancer, Lung Adenocarcinoma	[37-46]
miR-452-5p	Intronic (embedded within GABRE)	↓73 (BCC vs. HK)<br><br>↓152 (BCC vs. SCC)	↑Hemangiosarcoma, Clear Cell Renal Cell Carcinoma, bladder cancer	[47-56]

			↓Lung adenocarcinoma (mir-452-5p), chondrosarcoma, gliomas, osteosarcoma, non-small cell lung cancer, prostate cancer, head and neck adenoid cystic carcinoma in comparison to head and neck squamous cell carcinoma	
miR-487b-3p	Part of MIR381 host gene (MIR381HG)	↓17 (BCC vs. HK)<br><br>↓31 (BCC vs. SCC)	↓Colon cancer (487b-3p), high-risk neuroblastoma, pediatric glioma, metastatic prostate cancer	[57-60]
miR-494-3p	One of a cluster on Chromosome 14	↓20 (BCC vs. HK)<br><br>↓20 (BCC vs. SCC)	↑Cervical cancer, colorectal cancer, Hepatocellular carcinoma, non-small cell lung cancer<br><br>↓Malignant breast cancer, esophageal squamous cell carcinoma, gastric carcinoma, oral cancer, ovarian cancer, pancreatic cancer	[61-70]
miR-590-5p	Intronic (embedded within EIF4H)	↓111 (BCC vs. HK)<br><br>↓391 (BCC vs. SCC)	↑Cervical cancer, gastric cancer, vulvar squamous cell carcinoma, renal cell carcinoma<br><br>↓Malignant melanoma, breast cancer, colorectal cancer	[71-77]
Differential expression BCC vs. HK				
miR-139-3p	Intronic (embedded within PDE2A)	↑144	↓Colorectal and bladder cancers, supraglottic laryngeal squamous cell carcinoma, Pancreatic ductal	[78-81]

			adenocarcinoma, breast cancer	
miR-302b	Anti-sense to LARP7, part of multi-miRNA transcript	↓56	↓High-grade gastric adenocarcinoma, gastric cancer, breast cancer, hepatocellular carcinoma, ovarian cancer, esophageal squamous cell carcinoma	[82-87]
miR-597	Intronic (embedded within TNKS)	↓72	↓Colorectal and breast cancer	[88, 89]
Differential expression SCC vs. HK				
miR-127-5p	intronic	↓207,965	↓Breast cancer, gastric cancer, hepatocellular carcinoma,<br>colon cancer patients' stool	[6, 90-92]
miR-135b	Intronic (embedded within BLACAT1)	↑100	↓ Gastric cancer, prostate cancer glioblastoma<br><br>↑ Gastric cancer mucosa, cervical cancer, colorectal cancer, non small cell lung cancer (highly invasive), glioblastoma	[93-102]
miR-187	Intergenic (chromosome 18)	↑45	↑ Gastric cancer, Oral squamous cell carcinoma, ovary clear cell carcinoma<br><br>↓ Colorectal cancer, clear cell renal cell carcinoma, gallbladder cancer, prostate cancer	[93, 103-109]
miR-493	Intergenic (chromosome 14)	↑38	↑Gastric cancer (enhances invasion)<br><br>↓Liver metastatic colon cancer (antimetastatic effect)	[110, 111]

Differential expression SCC vs. BCC (invasion and metastasis associated)				
let-7e	Intronic (embedded within SPACA6)	↑32	↓Bronchioloalveolar carcinomas, lung cancer, breast cancer, colon cancer, esophageal squamous cell carcinoma, glioblastoma, nasopharyngeal carcinoma cells, pancreatic adenocarcinoma<br><br>↑Papillary thyroid carcinomas	[112-121]
miR-15a-5p	Intronic (embedded within DLEU2)	↑120	↑Esophageal cancer, glioma, neuroblastoma, colorectal cancer<br><br>↓ Breast cancer, B-cell Chronic lymphocytic leukemia, pituitary adenomas, Prostate cancer, colorectal cancer, gastric cancer, non-small cell lung cancer, osteosarcoma, pancreatic cancer, prostate cancer, squamous cell carcinomas and adenocarcinomas of the lung, hepatocellular carcinoma	[122-137]
miR-95-3p	Intronic (embedded within ABLIM2)	↑269	↑Colorectal cancer, Non-small cell lung cancer, head and neck cancer, pancreatic cancer, Hepatocellular carcinoma, glioma	[138-143]
miR-362-5p	Intronic (One of a cluster of miRNAs in CLCN5)	↑125	↑Chronic myeloid leukemia, hepatocellular carcinoma, gastric cancer<br><br>↓Cervical cancer, renal cell carcinoma, BRAF-	[144-153]

			mutated colorectal cancers<br>↓(miR-362-5p) ALK-positive non-small cell lung cancer,<br>breast cancer,<br>neuroblastoma,<br>colorectal cancer	
miR-502-5p	Intronic (One of a cluster of miRNAs in CLCN5)	↑66	↑Conjunctival malignant melanoma, Merkel cell carcinoma<br><br>↓Colon cancer, breast cancer	[154-158]
miR-518e	Intergenic (chromosome 19)	↑56	↑Hepatocellular carcinoma	[159, 160]
miR-520f -3p	Intergenic (chromosome 19)	↑7973	↑Glioblastoma Tumor Cells<br><br>↓Gastric carcinoma, neuroblastoma	[161-163]
miR-886-3p	Intergenic (chromosome 5)	↑13	↑ALK-positive Anaplastic large-cell lymphomas<br><br>↓Papillary thyroid, Small-cell<br>lung cancer,<br>squamous cell lung carcinoma	[164-167]

References:

1. Zhang Z, Li Y, Fan L, Zhao Q, Tan B, Li Z, et al. microRNA-425-5p is upregulated in human gastric cancer and contributes to invasion and metastasis in vitro and in vivo. Exp Ther Med. 2015;9(5):1617-22. doi: 10.3892/etm.2015.2318. PubMed PMID: 26136868; PubMed Central PMCID: PMCPMC4471687.
2. Ueda T, Volinia S, Okumura H, Shimizu M, Taccioli C, Rossi S, et al. Relation between microRNA expression and progression and prognosis of gastric cancer: a microRNA expression analysis. Lancet Oncol. 2010;11(2):136-46. doi: 10.1016/S1470-2045(09)70343-2. PubMed PMID: 20022810; PubMed Central PMCID: PMCPMC4299826.
3. Fleming NH, Zhong J, da Silva IP, Vega-Saenz de Miera E, Brady B, Han SW, et al. Serum-based miRNAs in the prediction and detection of recurrence in melanoma

patients. Cancer. 2015;121(1):51-9. doi: 10.1002/cncr.28981. PubMed PMID: 25155861; PubMed Central PMCID: PMCPMC4270907.

4. Weiner-Gorzel K, Dempsey E, Milewska M, McGoldrick A, Toh V, Walsh A, et al. Overexpression of the microRNA miR-433 promotes resistance to paclitaxel through the induction of cellular senescence in ovarian cancer cells. Cancer Med. 2015;4(5):745-58. doi: 10.1002/cam4.409. PubMed PMID: 25684390; PubMed Central PMCID: PMCPMC4430267.
5. Yang Z, Tsuchiya H, Zhang Y, Hartnett ME, Wang L. MicroRNA-433 inhibits liver cancer cell migration by repressing the protein expression and function of cAMP response element-binding protein. J Biol Chem. 2013;288(40):28893-9. doi: 10.1074/jbc.M113.502682. PubMed PMID: 23979134; PubMed Central PMCID: PMCPMC3789984.
6. Guo LH, Li H, Wang F, Yu J, He JS. The Tumor Suppressor Roles of miR-433 and miR-127 in Gastric Cancer. Int J Mol Sci. 2013;14(7):14171-84. doi: 10.3390/ijms140714171. PubMed PMID: 23880861; PubMed Central PMCID: PMCPMC3742237.
7. Xu X, Zhu Y, Liang Z, Li S, Xu X, Wang X, et al. c-Met and CREB1 are involved in miR-433-mediated inhibition of the epithelial-mesenchymal transition in bladder cancer by regulating Akt/GSK-3beta/Snail signaling. Cell Death Dis. 2016;7:e2088. doi: 10.1038/cddis.2015.274. PubMed PMID: 26844702; PubMed Central PMCID: PMCPMC4849142.
8. Furlong F, Fitzpatrick P, O'Toole S, Phelan S, McGrogan B, Maguire A, et al. Low MAD2 expression levels associate with reduced progression-free survival in patients with high-grade serous epithelial ovarian cancer. J Pathol. 2012;226(5):746-55. doi: 10.1002/path.3035. PubMed PMID: 22069160; PubMed Central PMCID: PMCPMC3593171.
9. Schaefer A, Jung M, Mollenkopf HJ, Wagner I, Stephan C, Jentzmik F, et al. Diagnostic and prognostic implications of microRNA profiling in prostate carcinoma. Int J Cancer. 2010;126(5):1166-76. doi: 10.1002/ijc.24827. PubMed PMID: 19676045.
10. Wong TS, Liu XB, Wong BY, Ng RW, Yuen AP, Wei WI. Mature miR-184 as Potential Oncogenic microRNA of Squamous Cell Carcinoma of Tongue. Clin Cancer Res. 2008;14(9):2588-92. doi: 10.1158/1078-0432.CCR-07-0666. PubMed PMID: 18451220.
11. Fu L, Li Z, Zhu J, Wang P, Fan G, Dai Y, et al. Serum expression levels of microRNA-382-3p, -598-3p, -1246 and -184 in breast cancer patients. Oncol Lett. 2016;12(1):269-74. doi: 10.3892/ol.2016.4582. PubMed PMID: 27347136; PubMed Central PMCID: PMCPMC4906595.
12. Cui QK, Liu WD, Zhu JX, Wang YH, Wang ZG. MicroRNA-184 promotes proliferation ability of glioma cells by regulating FOXO3. Asian Pac J Trop Med. 2014;7(10):776-9. doi: 10.1016/S1995-7645(14)60135-8. PubMed PMID: 25129459.
13. Hsu CM, Lin PM, Wang YM, Chen ZJ, Lin SF, Yang MY. Circulating miRNA is a novel marker for head and neck squamous cell carcinoma. Tumour Biol. 2012;33(6):1933-42. doi: 10.1007/s13277-012-0454-8. PubMed PMID: 22811001.
14. Foley NH, Bray IM, Tivnan A, Bryan K, Murphy DM, Buckley PG, et al. MicroRNA-184 inhibits neuroblastoma cell survival through targeting the serine/threonine kinase AKT2. Mol Cancer. 2010;9:83. doi: 10.1186/1476-4598-9-83. PubMed PMID: 20409325; PubMed Central PMCID: PMCPMC2864218.

15. Loudig O, Wang T, Ye K, Lin J, Wang Y, Ramnauth A, et al. Evaluation and Adaptation of a Laboratory-Based cDNA Library Preparation Protocol for Retrospective Sequencing of Archived MicroRNAs from up to 35-Year-Old Clinical FFPE Specimens. Int J Mol Sci. 2017;18(3). doi: 10.3390/ijms18030627. PubMed PMID: 28335433; PubMed Central PMCID: PMCPMC5372640.
16. Qin CZ, Lou XY, Lv QL, Cheng L, Wu NY, Hu L, et al. MicroRNA-184 acts as a potential diagnostic and prognostic marker in epithelial ovarian cancer and regulates cell proliferation, apoptosis and inflammation. Pharmazie. 2015;70(10):668-73. PubMed PMID: 26601424.
17. Tetzlaff MT, Curry JL, Yin V, Pattanaprichakul P, Manonukul J, Uiprasertkul M, et al. Distinct pathways in the pathogenesis of sebaceous carcinomas implicated by differentially expressed microRNAs. JAMA Ophthalmol. 2015;133(10):1109-16. doi: 10.1001/jamaophthalmol.2015.2310. PubMed PMID: 26203913.
18. Lin TC, Lin PL, Cheng YW, Wu TC, Chou MC, Chen CY, et al. MicroRNA-184 Deregulated by the MicroRNA-21 Promotes Tumor Malignancy and Poor Outcomes in Non-small Cell Lung Cancer via Targeting CDC25A and c-Myc. Ann Surg Oncol. 2015;22 Suppl 3:S1532-9. doi: 10.1245/s10434-015-4595-z. PubMed PMID: 25990966.
19. Zhou R, Zhou X, Yin Z, Guo J, Hu T, Jiang S, et al. Tumor invasion and metastasis regulated by microRNA-184 and microRNA-574-5p in small-cell lung cancer. Oncotarget. 2015;6(42):44609-22. doi: 10.18632/oncotarget.6338. PubMed PMID: 26587830; PubMed Central PMCID: PMCPMC4792579.
20. Su Z, Chen D, Li Y, Zhang E, Yu Z, Chen T, et al. microRNA-184 functions as tumor suppressor in renal cell carcinoma. Exp Ther Med. 2015;9(3):961-6. doi: 10.3892/etm.2015.2199. PubMed PMID: 25667660; PubMed Central PMCID: PMCPMC4316952.
21. Gao B, Gao K, Li L, Huang Z, Lin L. miR-184 functions as an oncogenic regulator in hepatocellular carcinoma (HCC). Biomed Pharmacother. 2014;68(2):143-8. doi: 10.1016/j.biopha.2013.09.005. PubMed PMID: 24183204.
22. Wu GG, Li WH, He WG, Jiang N, Zhang GX, Chen W, et al. Mir-184 post-transcriptionally regulates SOX7 expression and promotes cell proliferation in human hepatocellular carcinoma. PLoS One. 2014;9(2):e88796. doi: 10.1371/journal.pone.0088796. PubMed PMID: 24558429; PubMed Central PMCID: PMCPMC3928300.
23. Liang Z, Li S, Xu X, Xu X, Wang X, Wu J, et al. MicroRNA-576-3p inhibits proliferation in bladder cancer cells by targeting cyclin D1. Mol Cells. 2015;38(2):130-7. doi: 10.14348/molcells.2015.2146. PubMed PMID: 25556372; PubMed Central PMCID: PMCPMC4332027.
24. Balci S, Ayaz L, Gorur A, Yildirim Yaroglu H, Akbayir S, Dogruer Unal N, et al. microRNA profiling for early detection of nonmelanoma skin cancer. Clin Exp Dermatol. 2016;41(4):346-51. doi: 10.1111/ced.12736. PubMed PMID: 26299703.
25. Coskun E, Neumann M, Schlee C, Liebertz F, Heesch S, Goekbuget N, et al. MicroRNA profiling reveals aberrant microRNA expression in adult ETP-ALL and functional studies implicate a role for miR-222 in acute leukemia. Leuk Res. 2013;37(6):647-56. doi: 10.1016/j.leukres.2013.02.019. PubMed PMID: 23522449.
26. Fan Y, Song X, Du H, Luo C, Wang X, Yang X, et al. Down-regulation of miR-29c in human bladder cancer and the inhibition of proliferation in T24 cell via PI3K-AKT

pathway. Med Oncol. 2014;31(7):65. doi: 10.1007/s12032-014-0065-x. PubMed PMID: 24952510.
27. Ding DP, Chen ZL, Zhao XH, Wang JW, Sun J, Wang Z, et al. miR-29c induces cell cycle arrest in esophageal squamous cell carcinoma by modulating cyclin E expression. Carcinogenesis. 2011;32(7):1025-32. doi: 10.1093/carcin/bgr078. PubMed PMID: 21551130.
28. Han TS, Hur K, Xu G, Choi B, Okugawa Y, Toiyama Y, et al. MicroRNA-29c mediates initiation of gastric carcinogenesis by directly targeting ITGB1. Gut. 2015;64(2):203-14. doi: 10.1136/gutjnl-2013-306640. PubMed PMID: 24870620; PubMed Central PMCID: PMCPMC4384419.
29. Fan YC, Mei PJ, Chen C, Miao FA, Zhang H, Li ZL. MiR-29c inhibits glioma cell proliferation, migration, invasion and angiogenesis. J Neurooncol. 2013;115(2):179-88. doi: 10.1007/s11060-013-1223-2. PubMed PMID: 23943502.
30. Kinoshita T, Nohata N, Hanazawa T, Kikkawa N, Yamamoto N, Yoshino H, et al. Tumour-suppressive microRNA-29s inhibit cancer cell migration and invasion by targeting laminin-integrin signalling in head and neck squamous cell carcinoma. British journal of cancer. 2013;109(10):2636-45. doi: 10.1038/bjc.2013.607. PubMed PMID: 24091622; PubMed Central PMCID: PMCPMC3833206.
31. Dong CW, Wang YX, Du FT, Ding W, Hu SY. Low miR-29c expression is a prognostic marker in hepatocellular carcinoma. Genet Mol Res. 2016;15(3). doi: 10.4238/gmr.15037316. PubMed PMID: 27525839.
32. Liu L, Bi N, Wu L, Ding X, Men Y, Zhou W, et al. MicroRNA-29c functions as a tumor suppressor by targeting VEGFA in lung adenocarcinoma. Mol Cancer. 2017;16(1):50. doi: 10.1186/s12943-017-0620-0. PubMed PMID: 28241836; PubMed Central PMCID: PMCPMC5330136.
33. Sengupta S, den Boon JA, Chen IH, Newton MA, Stanhope SA, Cheng YJ, et al. MicroRNA 29c is down-regulated in nasopharyngeal carcinomas, up-regulating mRNAs encoding extracellular matrix proteins. Proc Natl Acad Sci U S A. 2008;105(15):5874-8. doi: 10.1073/pnas.0801130105. PubMed PMID: 18390668; PubMed Central PMCID: PMCPMC2311339.
34. Wang H, Zhu Y, Zhao M, Wu C, Zhang P, Tang L, et al. miRNA-29c suppresses lung cancer cell adhesion to extracellular matrix and metastasis by targeting integrin beta1 and matrix metalloproteinase2 (MMP2). PLoS One. 2013;8(8):e70192. doi: 10.1371/journal.pone.0070192. PubMed PMID: 23936390; PubMed Central PMCID: PMCPMC3735565.
35. Heegaard NH, Schetter AJ, Welsh JA, Yoneda M, Bowman ED, Harris CC. Circulating micro-RNA expression profiles in early stage nonsmall cell lung cancer. Int J Cancer. 2012;130(6):1378-86. doi: 10.1002/ijc.26153. PubMed PMID: 21544802; PubMed Central PMCID: PMCPMC3259258.
36. Kwon JJ, Willy JA, Quirin KA, Wek RC, Korc M, Yin XM, et al. Novel role of miR-29a in pancreatic cancer autophagy and its therapeutic potential. Oncotarget. 2016;7(44):71635-50. doi: 10.18632/oncotarget.11928. PubMed PMID: 27626694; PubMed Central PMCID: PMCPMC5342107.
37. Yang X, Ruan H, Hu X, Cao A, Song L. miR-381-3p suppresses the proliferation of oral squamous cell carcinoma cells by directly targeting FGFR2. Am J Cancer Res.

2017;7(4):913-22. PubMed PMID: 28469963; PubMed Central PMCID: PMCPMC5411798.
38. Xia B, Li H, Yang S, Liu T, Lou G. MiR-381 inhibits epithelial ovarian cancer malignancy via YY1 suppression. Tumour Biol. 2016;37(7):9157-67. doi: 10.1007/s13277-016-4805-8. PubMed PMID: 26768613.
39. Zhang Q, Zhao S, Pang X, Chi B. MicroRNA-381 suppresses cell growth and invasion by targeting the liver receptor homolog-1 in hepatocellular carcinoma. Oncol Rep. 2016;35(3):1831-40. doi: 10.3892/or.2015.4491. PubMed PMID: 26677080.
40. He X, Wei Y, Wang Y, Liu L, Wang W, Li N. MiR-381 functions as a tumor suppressor in colorectal cancer by targeting Twist1. Onco Targets Ther. 2016;9:1231-9. doi: 10.2147/OTT.S99228. PubMed PMID: 27094913; PubMed Central PMCID: PMCPMC4789845.
41. Cao Q, Liu F, Ji K, Liu N, He Y, Zhang W, et al. MicroRNA-381 inhibits the metastasis of gastric cancer by targeting TMEM16A expression. J Exp Clin Cancer Res. 2017;36(1):29. doi: 10.1186/s13046-017-0499-z. PubMed PMID: 28193228; PubMed Central PMCID: PMCPMC5307754.
42. Xue Y, Xu W, Zhao W, Wang W, Zhang D, Wu P. miR-381 inhibited breast cancer cells proliferation, epithelial-to-mesenchymal transition and metastasis by targeting CXCR4. Biomed Pharmacother. 2017;86:426-33. doi: 10.1016/j.biopha.2016.12.051. PubMed PMID: 28012397.
43. Li Y, Zhao C, Yu Z, Chen J, She X, Li P, et al. Low expression of miR-381 is a favorite prognosis factor and enhances the chemosensitivity of osteosarcoma. Oncotarget. 2016;7(42):68585-96. doi: 10.18632/oncotarget.11861. PubMed PMID: 27612424; PubMed Central PMCID: PMCPMC5356575.
44. Liang Y, Zhao Q, Fan L, Zhang Z, Tan B, Liu Y, et al. Down-regulation of MicroRNA-381 promotes cell proliferation and invasion in colon cancer through up-regulation of LRH-1. Biomed Pharmacother. 2015;75:137-41. doi: 10.1016/j.biopha.2015.07.020. PubMed PMID: 26320367.
45. Rothschild SI, Tschan MP, Jaggi R, Fey MF, Gugger M, Gautschi O. MicroRNA-381 represses ID1 and is deregulated in lung adenocarcinoma. J Thorac Oncol. 2012;7(7):1069-77. doi: 10.1097/JTO.0b013e31824fe976. PubMed PMID: 22592211.
46. Tang H, Liu X, Wang Z, She X, Zeng X, Deng M, et al. Interaction of hsa-miR-381 and glioma suppressor LRRC4 is involved in glioma growth. Brain Res. 2011;1390:21-32. doi: 10.1016/j.brainres.2011.03.034. PubMed PMID: 21435336.
47. Gan XN, Luo J, Tang RX, Wang HL, Zhou H, Qin H, et al. Clinical value of miR-452-5p expression in lung adenocarcinoma: A retrospective quantitative real-time polymerase chain reaction study and verification based on The Cancer Genome Atlas and Gene Expression Omnibus databases. Tumour Biol. 2017;39(5):1010428317705755. doi: 10.1177/1010428317705755. PubMed PMID: 28488527.
48. Lin CY, Tzeng HE, Li TM, Chen HT, Lee Y, Yang YC, et al. WISP-3 inhibition of miR-452 promotes VEGF-A expression in chondrosarcoma cells and induces endothelial progenitor cells angiogenesis. Oncotarget. 2017. doi: 10.18632/oncotarget.17142. PubMed PMID: 28465477.
49. Grimes JA, Prasad N, Levy S, Cattley R, Lindley S, Boothe HW, et al. A comparison of microRNA expression profiles from splenic hemangiosarcoma, splenic nodular hyperplasia, and normal spleens of dogs. BMC Vet Res. 2016;12(1):272. doi:

10.1186/s12917-016-0903-5. PubMed PMID: 27912752; PubMed Central PMCID: PMCPMC5135805.
50. Zheng Z, Liu J, Yang Z, Wu L, Xie H, Jiang C, et al. MicroRNA-452 promotes stem-like cells of hepatocellular carcinoma by inhibiting Sox7 involving Wnt/beta-catenin signaling pathway. Oncotarget. 2016;7(19):28000-12. doi: 10.18632/oncotarget.8584. PubMed PMID: 27058905; PubMed Central PMCID: PMCPMC5053705.
51. Li RZ, Wang LM. Decreased microRNA-452 expression and its prognostic significance in human osteosarcoma. World J Surg Oncol. 2016;14:150. doi: 10.1186/s12957-016-0900-y. PubMed PMID: 27193084; PubMed Central PMCID: PMCPMC4870789.
52. He Z, Xia Y, Liu B, Qi X, Li Z, Wang J, et al. Down-regulation of miR-452 is associated with poor prognosis in the non-small-cell lung cancer. J Thorac Dis. 2016;8(5):894-900. doi: 10.21037/jtd.2016.03.51. PubMed PMID: 27162664; PubMed Central PMCID: PMCPMC4842820.
53. Goto Y, Kojima S, Kurozumi A, Kato M, Okato A, Matsushita R, et al. Regulation of E3 ubiquitin ligase-1 (WWP1) by microRNA-452 inhibits cancer cell migration and invasion in prostate cancer. British journal of cancer. 2016;114(10):1135-44. doi: 10.1038/bjc.2016.95. PubMed PMID: 27070713; PubMed Central PMCID: PMCPMC4865980.
54. He H, Wang L, Zhou W, Zhang Z, Wang L, Xu S, et al. MicroRNA Expression Profiling in Clear Cell Renal Cell Carcinoma: Identification and Functional Validation of Key miRNAs. PLoS One. 2015;10(5):e0125672. doi: 10.1371/journal.pone.0125672. PubMed PMID: 25938468; PubMed Central PMCID: PMCPMC4418764.
55. Veit JA, Scheckenbach K, Schuler PJ, Laban S, Wiggenhauser PS, Thierauf J, et al. MicroRNA expression in differentially metastasizing tumors of the head and neck: adenoid cystic versus squamous cell carcinoma. Anticancer Res. 2015;35(3):1271-7. PubMed PMID: 25750274.
56. Puerta-Gil P, Garcia-Baquero R, Jia AY, Ocana S, Alvarez-Mugica M, Alvarez-Ossorio JL, et al. miR-143, miR-222, and miR-452 are useful as tumor stratification and noninvasive diagnostic biomarkers for bladder cancer. Am J Pathol. 2012;180(5):1808-15. doi: 10.1016/j.ajpath.2012.01.034. PubMed PMID: 22426337.
57. Yi H, Geng L, Black A, Talmon G, Berim L, Wang J. The miR-487b-3p/GRM3/TGFbeta signaling axis is an important regulator of colon cancer tumorigenesis. Oncogene. 2017;36(24):3477-89. doi: 10.1038/onc.2016.499. PubMed PMID: 28114282.
58. Gattolliat CH, Thomas L, Ciafre SA, Meurice G, Le Teuff G, Job B, et al. Expression of miR-487b and miR-410 encoded by 14q32.31 locus is a prognostic marker in neuroblastoma. British journal of cancer. 2011;105(9):1352-61. doi: 10.1038/bjc.2011.388. PubMed PMID: 21970883; PubMed Central PMCID: PMCPMC3241557.
59. Ames HM, Yuan M, Vizcaino MA, Yu W, Rodriguez FJ. MicroRNA profiling of low-grade glial and glioneuronal tumors shows an independent role for cluster 14q32.31 member miR-487b. Mod Pathol. 2017;30(2):204-16. doi: 10.1038/modpathol.2016.177. PubMed PMID: 27739438; PubMed Central PMCID: PMCPMC5288128.
60. Formosa A, Markert EK, Lena AM, Italiano D, Finazzi-Agro E, Levine AJ, et al. MicroRNAs, miR-154, miR-299-5p, miR-376a, miR-376c, miR-377, miR-381, miR-487b, miR-485-3p, miR-495 and miR-654-3p, mapped to the 14q32.31 locus, regulate proliferation, apoptosis, migration and invasion in metastatic prostate cancer cells.

Oncogene. 2014;33(44):5173-82. doi: 10.1038/onc.2013.451. PubMed PMID: 24166498.
61. Song L, Liu D, Wang B, He J, Zhang S, Dai Z, et al. miR-494 suppresses the progression of breast cancer in vitro by targeting CXCR4 through the Wnt/beta-catenin signaling pathway. Oncol Rep. 2015;34(1):525-31. doi: 10.3892/or.2015.3965. PubMed PMID: 25955111.
62. Yang YK, Xi WY, Xi RX, Li JY, Li Q, Gao YE. MicroRNA-494 promotes cervical cancer proliferation through the regulation of PTEN. Oncol Rep. 2015;33(5):2393-401. doi: 10.3892/or.2015.3821. PubMed PMID: 25738254.
63. Sun HB, Chen X, Ji H, Wu T, Lu HW, Zhang Y, et al. miR494 is an independent prognostic factor and promotes cell migration and invasion in colorectal cancer by directly targeting PTEN. Int J Oncol. 2014;45(6):2486-94. doi: 10.3892/ijo.2014.2665. PubMed PMID: 25270723.
64. Zhang R, Chen X, Zhang S, Zhang X, Li T, Liu Z, et al. Upregulation of miR-494 Inhibits Cell Growth and Invasion and Induces Cell Apoptosis by Targeting Cleft Lip and Palate Transmembrane 1-Like in Esophageal Squamous Cell Carcinoma. Dig Dis Sci. 2015;60(5):1247-55. doi: 10.1007/s10620-014-3433-7. PubMed PMID: 25480402.
65. He W, Li Y, Chen X, Lu L, Tang B, Wang Z, et al. miR-494 acts as an anti-oncogene in gastric carcinoma by targeting c-myc. J Gastroenterol Hepatol. 2014;29(7):1427-34. doi: 10.1111/jgh.12558. PubMed PMID: 24612089.
66. Lim L, Balakrishnan A, Huskey N, Jones KD, Jodari M, Ng R, et al. MicroRNA-494 within an oncogenic microRNA megacluster regulates G1/S transition in liver tumorigenesis through suppression of mutated in colorectal cancer. Hepatology. 2014;59(1):202-15. doi: 10.1002/hep.26662. PubMed PMID: 23913442; PubMed Central PMCID: PMCPMC3877416.
67. Wang J, Chen H, Liao Y, Chen N, Liu T, Zhang H, et al. Expression and clinical evidence of miR-494 and PTEN in non-small cell lung cancer. Tumour Biol. 2015;36(9):6965-72. doi: 10.1007/s13277-015-3416-0. PubMed PMID: 25861022.
68. Liborio-Kimura TN, Jung HM, Chan EK. miR-494 represses HOXA10 expression and inhibits cell proliferation in oral cancer. Oral Oncol. 2015;51(2):151-7. doi: 10.1016/j.oraloncology.2014.11.019. PubMed PMID: 25500095.
69. Kim YW, Kim EY, Jeon D, Liu JL, Kim HS, Choi JW, et al. Differential microRNA expression signatures and cell type-specific association with Taxol resistance in ovarian cancer cells. Drug Des Devel Ther. 2014;8:293-314. doi: 10.2147/DDDT.S51969. PubMed PMID: 24591819; PubMed Central PMCID: PMCPMC3938445.
70. Ma YB, Li GX, Hu JX, Liu X, Shi BM. Correlation of miR-494 expression with tumor progression and patient survival in pancreatic cancer. Genet Mol Res. 2015;14(4):18153-9. doi: 10.4238/2015.December.23.2. PubMed PMID: 26782462.
71. Wang L, Shi S, Zhou Y, Mu X, Han D, Ge R, et al. [miR-590-5p inhibits A375 cell invasion and migration in malignant melanoma by directly inhibiting YAP1 expression]. Xi Bao Yu Fen Zi Mian Yi Xue Za Zhi. 2017;33(3):326-30. PubMed PMID: 28274310.
72. Zhou L, Zhao LC, Jiang N, Wang XL, Zhou XN, Luo XL, et al. MicroRNA miR-590-5p inhibits breast cancer cell stemness and metastasis by targeting SOX2. Eur Rev Med Pharmacol Sci. 2017;21(1):87-94. PubMed PMID: 28121351.

73. Chu Y, Ouyang Y, Wang F, Zheng A, Bai L, Han L, et al. MicroRNA-590 promotes cervical cancer cell growth and invasion by targeting CHL1. J Cell Biochem. 2014;115(5):847-53. doi: 10.1002/jcb.24726. PubMed PMID: 24288179.
74. Zhou Q, Zhu Y, Wei X, Zhou J, Chang L, Sui H, et al. MiR-590-5p inhibits colorectal cancer angiogenesis and metastasis by regulating nuclear factor 90/vascular endothelial growth factor A axis. Cell Death Dis. 2016;7(10):e2413. doi: 10.1038/cddis.2016.306. PubMed PMID: 27735951; PubMed Central PMCID: PMCPMC5133975.
75. Shen B, Yu S, Zhang Y, Yuan Y, Li X, Zhong J, et al. miR-590-5p regulates gastric cancer cell growth and chemosensitivity through RECK and the AKT/ERK pathway. Onco Targets Ther. 2016;9:6009-19. doi: 10.2147/OTT.S110923. PubMed PMID: 27757042; PubMed Central PMCID: PMCPMC5055051.
76. Yang X, Wu X. miRNA expression profile of vulvar squamous cell carcinoma and identification of the oncogenic role of miR-590-5p. Oncol Rep. 2016;35(1):398-408. doi: 10.3892/or.2015.4344. PubMed PMID: 26498065.
77. Xiao X, Tang C, Xiao S, Fu C, Yu P. Enhancement of proliferation and invasion by MicroRNA-590-5p via targeting PBRM1 in clear cell renal carcinoma cells. Oncol Res. 2013;20(11):537-44. doi: 10.3727/096504013X13775486749335. PubMed PMID: 24063284.
78. Liu X, Duan B, Dong Y, He C, Zhou H, Sheng H, et al. MicroRNA-139-3p indicates a poor prognosis of colon cancer. Int J Clin Exp Pathol. 2014;7(11):8046-52. PubMed PMID: 25550849; PubMed Central PMCID: PMCPMC4270559.
79. Yonemori M, Seki N, Yoshino H, Matsushita R, Miyamoto K, Nakagawa M, et al. Dual tumor-suppressors miR-139-5p and miR-139-3p targeting matrix metalloprotease 11 in bladder cancer. Cancer Sci. 2016;107(9):1233-42. doi: 10.1111/cas.13002. PubMed PMID: 27355528; PubMed Central PMCID: PMCPMC5021030.
80. Sun X, Song Y, Tai X, Liu B, Ji W. MicroRNA expression and its detection in human supraglottic laryngeal squamous cell carcinoma. Biomed Rep. 2013;1(5):743-6. doi: 10.3892/br.2013.143. PubMed PMID: 24649021; PubMed Central PMCID: PMCPMC3917082.
81. Feliciano A, Castellvi J, Artero-Castro A, Leal JA, Romagosa C, Hernandez-Losa J, et al. miR-125b acts as a tumor suppressor in breast tumorigenesis via its novel direct targets ENPEP, CK2-alpha, CCNJ, and MEGF9. PLoS One. 2013;8(10):e76247. doi: 10.1371/journal.pone.0076247. PubMed PMID: 24098452; PubMed Central PMCID: PMCPMC3789742.
82. Khalili M, Sadeghizadeh M, Ghorbanian K, Malekzadeh R, Vasei M, Mowla SJ. Down-regulation of miR-302b, an ESC-specific microRNA, in Gastric Adenocarcinoma. Cell J. 2012;13(4):251-8. PubMed PMID: 23508453; PubMed Central PMCID: PMCPMC3584484.
83. Liu FY, Wang LP, Wang Q, Han P, Zhuang WP, Li MJ, et al. miR-302b regulates cell cycles by targeting CDK2 via ERK signaling pathway in gastric cancer. Cancer Med. 2016;5(9):2302-13. doi: 10.1002/cam4.818. PubMed PMID: 27465546; PubMed Central PMCID: PMCPMC5055145.
84. Cataldo A, Cheung DG, Balsari A, Tagliabue E, Coppola V, Iorio MV, et al. miR-302b enhances breast cancer cell sensitivity to cisplatin by regulating E2F1 and the cellular

DNA damage response. Oncotarget. 2016;7(1):786-97. doi: 10.18632/oncotarget.6381. PubMed PMID: 26623722; PubMed Central PMCID: PMCPMC4808033.
85. Wang L, Yao J, Zhang X, Guo B, Le X, Cubberly M, et al. miRNA-302b suppresses human hepatocellular carcinoma by targeting AKT2. Mol Cancer Res. 2014;12(2):190-202. doi: 10.1158/1541-7786.MCR-13-0411. PubMed PMID: 24337067.
86. Iorio MV, Visone R, Di Leva G, Donati V, Petrocca F, Casalini P, et al. MicroRNA signatures in human ovarian cancer. Cancer Res. 2007;67(18):8699-707. doi: 10.1158/0008-5472.CAN-07-1936. PubMed PMID: 17875710.
87. Zhang M, Yang Q, Zhang L, Zhou S, Ye W, Yao Q, et al. miR-302b is a potential molecular marker of esophageal squamous cell carcinoma and functions as a tumor suppressor by targeting ErbB4. J Exp Clin Cancer Res. 2014;33:10. doi: 10.1186/1756-9966-33-10. PubMed PMID: 24438167; PubMed Central PMCID: PMCPMC3898821.
88. Chen WC, Lin MS, Ye YL, Gao HJ, Song ZY, Shen XY. microRNA expression pattern and its alteration following celecoxib intervention in human colorectal cancer. Exp Ther Med. 2012;3(6):1039-48. doi: 10.3892/etm.2012.531. PubMed PMID: 22970014; PubMed Central PMCID: PMCPMC3438602.
89. He J, Mai J, Li Y, Chen L, Xu H, Zhu X, et al. miR-597 inhibits breast cancer cell proliferation, migration and invasion through FOSL2. Oncol Rep. 2017;37(5):2672-8. doi: 10.3892/or.2017.5558. PubMed PMID: 28393251; PubMed Central PMCID: PMCPMC5428280.
90. Pronina IV, Loginov VI, Burdennyy AM, Fridman MV, Senchenko VN, Kazubskaya TP, et al. DNA methylation contributes to deregulation of 12 cancer-associated microRNAs and breast cancer progression. Gene. 2017;604:1-8. doi: 10.1016/j.gene.2016.12.018. PubMed PMID: 27998789.
91. Zhou J, Lu S, Yang S, Chen H, Shi H, Miao M, et al. MicroRNA-127 post-transcriptionally downregulates Sept7 and suppresses cell growth in hepatocellular carcinoma cells. Cell Physiol Biochem. 2014;33(5):1537-46. doi: 10.1159/000358717. PubMed PMID: 24854842.
92. Ahmed FE, Ahmed NC, Vos PW, Bonnerup C, Atkins JN, Casey M, et al. Diagnostic microRNA markers to screen for sporadic human colon cancer in stool: I. Proof of principle. Cancer Genomics Proteomics. 2013;10(3):93-113. PubMed PMID: 23741026.
93. Lulli V, Buccarelli M, Martini M, Signore M, Biffoni M, Giannetti S, et al. miR-135b suppresses tumorigenesis in glioblastoma stem-like cells impairing proliferation, migration and self-renewal. Oncotarget. 2015;6(35):37241-56. doi: 10.18632/oncotarget.5925. PubMed PMID: 26437223; PubMed Central PMCID: PMCPMC4741927.
94. Chen MB, Liu YY, Cheng LB, Lu JW, Zeng P, Lu PH. AMPKalpha phosphatase Ppm1E upregulation in human gastric cancer is required for cell proliferation. Oncotarget. 2017;8(19):31288-96. doi: 10.18632/oncotarget.16126. PubMed PMID: 28423719; PubMed Central PMCID: PMCPMC5458207.
95. Lee JW, Kim N, Park JH, Kim HJ, Chang H, Kim JM, et al. Differential MicroRNA Expression Between Gastric Cancer Tissue and Non-cancerous Gastric Mucosa According to Helicobacter pylori Status. J Cancer Prev. 2017;22(1):33-9. doi: 10.15430/JCP.2017.22.1.33. PubMed PMID: 28382284; PubMed Central PMCID: PMCPMC5380187.

96. Xu Y, Zhao S, Cui M, Wang Q. Down-regulation of microRNA-135b inhibited growth of cervical cancer cells by targeting FOXO1. Int J Clin Exp Pathol. 2015;8(9):10294-304. PubMed PMID: 26617737; PubMed Central PMCID: PMCPMC4637552.
97. Faltejskova P, Svoboda M, Srutova K, Mlcochova J, Besse A, Nekvindova J, et al. Identification and functional screening of microRNAs highly deregulated in colorectal cancer. J Cell Mol Med. 2012;16(11):2655-66. doi: 10.1111/j.1582-4934.2012.01579.x. PubMed PMID: 22469014; PubMed Central PMCID: PMCPMC4118234.
98. Xu XM, Qian JC, Deng ZL, Cai Z, Tang T, Wang P, et al. Expression of miR-21, miR-31, miR-96 and miR-135b is correlated with the clinical parameters of colorectal cancer. Oncol Lett. 2012;4(2):339-45. doi: 10.3892/ol.2012.714. PubMed PMID: 22844381; PubMed Central PMCID: PMCPMC3402725.
99. Xue Y, Ni T, Jiang Y, Li Y. LncRNA GAS5 Inhibits Tumorigenesis and Enhances Radiosensitivity By Suppressing miR-135b Expression in Non-Small Cell Lung Cancer. Oncol Res. 2017. doi: 10.3727/096504017X14850182723737. PubMed PMID: 28117028.
100. Lin CW, Chang YL, Chang YC, Lin JC, Chen CC, Pan SH, et al. MicroRNA-135b promotes lung cancer metastasis by regulating multiple targets in the Hippo pathway and LZTS1. Nat Commun. 2013;4:1877. doi: 10.1038/ncomms2876. PubMed PMID: 23695671.
101. Wang N, Tao L, Zhong H, Zhao S, Yu Y, Yu B, et al. miR-135b inhibits tumour metastasis in prostate cancer by targeting STAT6. Oncol Lett. 2016;11(1):543-50. doi: 10.3892/ol.2015.3970. PubMed PMID: 26870245; PubMed Central PMCID: PMCPMC4727074.
102. Yu Y, Zhang JH, Zhang BZ. MicroRNA-135b exerts oncogenic activity in glioblastoma via the inhibition of glycerol kinase 5 expression. Mol Med Rep. 2015;12(2):2721-6. doi: 10.3892/mmr.2015.3708. PubMed PMID: 25936394.
103. Zhang F, Luo Y, Shao Z, Xu L, Liu X, Niu Y, et al. MicroRNA-187, a downstream effector of TGFbeta pathway, suppresses Smad-mediated epithelial-mesenchymal transition in colorectal cancer. Cancer Lett. 2016;373(2):203-13. doi: 10.1016/j.canlet.2016.01.037. PubMed PMID: 26820227.
104. Ren L, Li F, Di M, Fu Y, Hui Y, Xiao G, et al. MicroRNA-187 regulates gastric cancer progression by targeting the tumor suppressor CRMP1. Biochem Biophys Res Commun. 2017;482(4):597-603. doi: 10.1016/j.bbrc.2016.11.079. PubMed PMID: 27864146.
105. Li C, Lu S, Shi Y. MicroRNA-187 promotes growth and metastasis of gastric cancer by inhibiting FOXA2. Oncol Rep. 2017;37(3):1747-55. doi: 10.3892/or.2017.5370. PubMed PMID: 28098868.
106. Lin SC, Kao SY, Chang JC, Liu YC, Yu EH, Tseng SH, et al. Up-regulation of miR-187 modulates the advances of oral carcinoma by targeting BARX2 tumor suppressor. Oncotarget. 2016;7(38):61355-65. doi: 10.18632/oncotarget.11349. PubMed PMID: 27542258; PubMed Central PMCID: PMCPMC5308656.
107. Zhao J, Lei T, Xu C, Li H, Ma W, Yang Y, et al. MicroRNA-187, down-regulated in clear cell renal cell carcinoma and associated with lower survival, inhibits cell growth and migration though targeting B7-H3. Biochem Biophys Res Commun. 2013;438(2):439-44. doi: 10.1016/j.bbrc.2013.07.095. PubMed PMID: 23916610.

108. Chao A, Lai CH, Chen HC, Lin CY, Tsai CL, Tang YH, et al. Serum microRNAs in clear cell carcinoma of the ovary. Taiwan J Obstet Gynecol. 2014;53(4):536-41. doi: 10.1016/j.tjog.2014.07.005. PubMed PMID: 25510697.
109. Fuse M, Kojima S, Enokida H, Chiyomaru T, Yoshino H, Nohata N, et al. Tumor suppressive microRNAs (miR-222 and miR-31) regulate molecular pathways based on microRNA expression signature in prostate cancer. J Hum Genet. 2012;57(11):691-9. doi: 10.1038/jhg.2012.95. PubMed PMID: 22854542.
110. Jia X, Li N, Peng C, Deng Y, Wang J, Deng M, et al. miR-493 mediated DKK1 down-regulation confers proliferation, invasion and chemo-resistance in gastric cancer cells. Oncotarget. 2016;7(6):7044-54. doi: 10.18632/oncotarget.6951. PubMed PMID: 26799283; PubMed Central PMCID: PMCPMC4872767.
111. Okamoto K, Ishiguro T, Midorikawa Y, Ohata H, Izumiya M, Tsuchiya N, et al. miR-493 induction during carcinogenesis blocks metastatic settlement of colon cancer cells in liver. EMBO J. 2012;31(7):1752-63. doi: 10.1038/emboj.2012.25. PubMed PMID: 22373578; PubMed Central PMCID: PMCPMC3321205.
112. Yan Y, Zhang F, Fan Q, Li X, Zhou K. Breast cancer-specific TRAIL expression mediated by miRNA response elements of let-7 and miR-122. Neoplasma. 2014;61(6):672-9. doi: 10.4149/neo_2014_082. PubMed PMID: 25150312.
113. Inamura K, Togashi Y, Nomura K, Ninomiya H, Hiramatsu M, Satoh Y, et al. let-7 microRNA expression is reduced in bronchioloalveolar carcinoma, a non-invasive carcinoma, and is not correlated with prognosis. Lung Cancer. 2007;58(3):392-6. doi: 10.1016/j.lungcan.2007.07.013. PubMed PMID: 17728006.
114. Akao Y, Nakagawa Y, Naoe T. let-7 microRNA functions as a potential growth suppressor in human colon cancer cells. Biol Pharm Bull. 2006;29(5):903-6. PubMed PMID: 16651716.
115. He FC, Meng WW, Qu YH, Zhou MX, He J, Lv P, et al. Expression of circulating microRNA-20a and let-7a in esophageal squamous cell carcinoma. World J Gastroenterol. 2015;21(15):4660-5. doi: 10.3748/wjg.v21.i15.4660. PubMed PMID: 25914476; PubMed Central PMCID: PMCPMC4402314.
116. Gong W, Zheng J, Liu X, Ma J, Liu Y, Xue Y. Knockdown of NEAT1 restrained the malignant progression of glioma stem cells by activating microRNA let-7e. Oncotarget. 2016;7(38):62208-23. doi: 10.18632/oncotarget.11403. PubMed PMID: 27556696; PubMed Central PMCID: PMCPMC5308721.
117. Takamizawa J, Konishi H, Yanagisawa K, Tomida S, Osada H, Endoh H, et al. Reduced expression of the let-7 microRNAs in human lung cancers in association with shortened postoperative survival. Cancer Res. 2004;64(11):3753-6. doi: 10.1158/0008-5472.CAN-04-0637. PubMed PMID: 15172979.
118. Karube Y, Tanaka H, Osada H, Tomida S, Tatematsu Y, Yanagisawa K, et al. Reduced expression of Dicer associated with poor prognosis in lung cancer patients. Cancer Sci. 2005;96(2):111-5. doi: 10.1111/j.1349-7006.2005.00015.x. PubMed PMID: 15723655.
119. Yu S, Liu Y, Wang J, Guo Z, Zhang Q, Yu F, et al. Circulating microRNA profiles as potential biomarkers for diagnosis of papillary thyroid carcinoma. J Clin Endocrinol Metab. 2012;97(6):2084-92. doi: 10.1210/jc.2011-3059. PubMed PMID: 22472564.
120. Wong TS, Man OY, Tsang CM, Tsao SW, Tsang RK, Chan JY, et al. MicroRNA let-7 suppresses nasopharyngeal carcinoma cells proliferation through downregulating c-Myc

expression. J Cancer Res Clin Oncol. 2011;137(3):415-22. doi: 10.1007/s00432-010-0898-4. PubMed PMID: 20440510; PubMed Central PMCID: PMCPMC3036828.
121. Torrisani J, Bournet B, du Rieu MC, Bouisson M, Souque A, Escourrou J, et al. let-7 MicroRNA transfer in pancreatic cancer-derived cells inhibits in vitro cell proliferation but fails to alter tumor progression. Hum Gene Ther. 2009;20(8):831-44. doi: 10.1089/hum.2008.134. PubMed PMID: 19323605.
122. Luo Q, Li X, Li J, Kong X, Zhang J, Chen L, et al. MiR-15a is underexpressed and inhibits the cell cycle by targeting CCNE1 in breast cancer. Int J Oncol. 2013;43(4):1212-8. doi: 10.3892/ijo.2013.2034. PubMed PMID: 23900351.
123. Calin GA, Croce CM. Genomics of chronic lymphocytic leukemia microRNAs as new players with clinical significance. Semin Oncol. 2006;33(2):167-73. doi: 10.1053/j.seminoncol.2006.01.010. PubMed PMID: 16616063.
124. Bottoni A, Piccin D, Tagliati F, Luchin A, Zatelli MC, degli Uberti EC. miR-15a and miR-16-1 down-regulation in pituitary adenomas. J Cell Physiol. 2005;204(1):280-5. doi: 10.1002/jcp.20282. PubMed PMID: 15648093.
125. Bonci D, Coppola V, Musumeci M, Addario A, Giuffrida R, Memeo L, et al. The miR-15a-miR-16-1 cluster controls prostate cancer by targeting multiple oncogenic activities. Nat Med. 2008;14(11):1271-7. doi: 10.1038/nm.1880. PubMed PMID: 18931683.
126. Wang X, Wang J, Ma H, Zhang J, Zhou X. Downregulation of miR-195 correlates with lymph node metastasis and poor prognosis in colorectal cancer. Med Oncol. 2012;29(2):919-27. doi: 10.1007/s12032-011-9880-5. PubMed PMID: 21390519.
127. Liu SG, Qin XG, Zhao BS, Qi B, Yao WJ, Wang TY, et al. Differential expression of miRNAs in esophageal cancer tissue. Oncol Lett. 2013;5(5):1639-42. doi: 10.3892/ol.2013.1251. PubMed PMID: 23761828; PubMed Central PMCID: PMCPMC3678876.
128. Wu C, Zheng X, Li X, Fesler A, Hu W, Chen L, et al. Reduction of gastric cancer proliferation and invasion by miR-15a mediated suppression of Bmi-1 translation. Oncotarget. 2016;7(12):14522-36. doi: 10.18632/oncotarget.7392. PubMed PMID: 26894855; PubMed Central PMCID: PMCPMC4924733.
129. Ye X, Wei W, Zhang Z, He C, Yang R, Zhang J, et al. Identification of microRNAs associated with glioma diagnosis and prognosis. Oncotarget. 2017;8(16):26394-403. doi: 10.18632/oncotarget.14445. PubMed PMID: 28060761; PubMed Central PMCID: PMCPMC5432266.
130. Yang T, Thakur A, Chen T, Yang L, Lei G, Liang Y, et al. MicroRNA-15a induces cell apoptosis and inhibits metastasis by targeting BCL2L2 in non-small cell lung cancer. Tumour Biol. 2015;36(6):4357-65. doi: 10.1007/s13277-015-3075-1. PubMed PMID: 25874488.
131. Tian X, Zhang J, Yan L, Dong JM, Guo Q. MiRNA-15a inhibits proliferation, migration and invasion by targeting TNFAIP1 in human osteosarcoma cells. Int J Clin Exp Pathol. 2015;8(6):6442-9. PubMed PMID: 26261520; PubMed Central PMCID: PMCPMC4525854.
132. Zhang XJ, Ye H, Zeng CW, He B, Zhang H, Chen YQ. Dysregulation of miR-15a and miR-214 in human pancreatic cancer. J Hematol Oncol. 2010;3:46. doi: 10.1186/1756-8722-3-46. PubMed PMID: 21106054; PubMed Central PMCID: PMCPMC3002909.
133. Guo S, Xu X, Tang Y, Zhang C, Li J, Ouyang Y, et al. miR-15a inhibits cell proliferation and epithelial to mesenchymal transition in pancreatic ductal adenocarcinoma by down-

regulating Bmi-1 expression. Cancer Lett. 2014;344(1):40-6. doi: 10.1016/j.canlet.2013.10.009. PubMed PMID: 24252251.
134. Xuan H, Xue W, Pan J, Sha J, Dong B, Huang Y. Downregulation of miR-221, -30d, and -15a contributes to pathogenesis of prostate cancer by targeting Bmi-1. Biochemistry (Mosc). 2015;80(3):276-83. doi: 10.1134/S0006297915030037. PubMed PMID: 25761682.
135. Bandi N, Zbinden S, Gugger M, Arnold M, Kocher V, Hasan L, et al. miR-15a and miR-16 are implicated in cell cycle regulation in a Rb-dependent manner and are frequently deleted or down-regulated in non-small cell lung cancer. Cancer Res. 2009;69(13):5553-9. doi: 10.1158/0008-5472.CAN-08-4277. PubMed PMID: 19549910.
136. de Groen FL, Timmer LM, Menezes RX, Diosdado B, Hooijberg E, Meijer GA, et al. Oncogenic Role of miR-15a-3p in 13q Amplicon-Driven Colorectal Adenoma-to-Carcinoma Progression. PLoS One. 2015;10(7):e0132495. doi: 10.1371/journal.pone.0132495. PubMed PMID: 26148070; PubMed Central PMCID: PMCPMC4492490.
137. Long J, Jiang C, Liu B, Fang S, Kuang M. MicroRNA-15a-5p suppresses cancer proliferation and division in human hepatocellular carcinoma by targeting BDNF. Tumour Biol. 2016;37(5):5821-8. doi: 10.1007/s13277-015-4427-6. PubMed PMID: 26581909.
138. Huang Z, Huang S, Wang Q, Liang L, Ni S, Wang L, et al. MicroRNA-95 promotes cell proliferation and targets sorting Nexin 1 in human colorectal carcinoma. Cancer Res. 2011;71(7):2582-9. doi: 10.1158/0008-5472.CAN-10-3032. PubMed PMID: 21427358.
139. Chen X, Chen S, Hang W, Huang H, Ma H. MiR-95 induces proliferation and chemo- or radioresistance through directly targeting sorting nexin1 (SNX1) in non-small cell lung cancer. Biomed Pharmacother. 2014;68(5):589-95. doi: 10.1016/j.biopha.2014.04.008. PubMed PMID: 24835695.
140. Ye J, Yao Y, Song Q, Li S, Hu Z, Yu Y, et al. Up-regulation of miR-95-3p in hepatocellular carcinoma promotes tumorigenesis by targeting p21 expression. Sci Rep. 2016;6:34034. doi: 10.1038/srep34034. PubMed PMID: 27698442; PubMed Central PMCID: PMCPMC5048429.
141. Fan B, Jiao BH, Fan FS, Lu SK, Song J, Guo CY, et al. Downregulation of miR-95-3p inhibits proliferation, and invasion promoting apoptosis of glioma cells by targeting CELF2. Int J Oncol. 2015;47(3):1025-33. doi: 10.3892/ijo.2015.3080. PubMed PMID: 26165303.
142. Nurul-Syakima AM, Yoke-Kqueen C, Sabariah AR, Shiran MS, Singh A, Learn-Han L. Differential microRNA expression and identification of putative miRNA targets and pathways in head and neck cancers. Int J Mol Med. 2011;28(3):327-36. doi: 10.3892/ijmm.2011.714. PubMed PMID: 21637912.
143. Zhang Y, Li M, Wang H, Fisher WE, Lin PH, Yao Q, et al. Profiling of 95 microRNAs in pancreatic cancer cell lines and surgical specimens by real-time PCR analysis. World J Surg. 2009;33(4):698-709. doi: 10.1007/s00268-008-9833-0. PubMed PMID: 19030927; PubMed Central PMCID: PMCPMC2933040.
144. Shi C, Zhang Z. MicroRNA-362 is downregulated in cervical cancer and inhibits cell proliferation, migration and invasion by directly targeting SIX1. Oncol Rep. 2017;37(1):501-9. doi: 10.3892/or.2016.5242. PubMed PMID: 27878258.

145. Wang C, Hu J, Lu M, Gu H, Zhou X, Chen X, et al. A panel of five serum miRNAs as a potential diagnostic tool for early-stage renal cell carcinoma. Sci Rep. 2015;5:7610. doi: 10.1038/srep07610. PubMed PMID: 25556603; PubMed Central PMCID: PMCPMC5154588.
146. Li LL, Qu LL, Fu HJ, Zheng XF, Tang CH, Li XY, et al. Circulating microRNAs as novel biomarkers of ALK-positive nonsmall cell lung cancer and predictors of response to crizotinib therapy. Oncotarget. 2017. doi: 10.18632/oncotarget.17535. PubMed PMID: 28514730.
147. Choi YW, Song YS, Lee H, Yi K, Kim YB, Suh KW, et al. MicroRNA Expression Signatures Associated With BRAF-Mutated Versus KRAS-Mutated Colorectal Cancers. Medicine (Baltimore). 2016;95(15):e3321. doi: 10.1097/MD.0000000000003321. PubMed PMID: 27082577; PubMed Central PMCID: PMCPMC4839821.
148. Ni F, Gui Z, Guo Q, Hu Z, Wang X, Chen D, et al. Downregulation of miR-362-5p inhibits proliferation, migration and invasion of human breast cancer MCF7 cells. Oncol Lett. 2016;11(2):1155-60. doi: 10.3892/ol.2015.3993. PubMed PMID: 26893711; PubMed Central PMCID: PMCPMC4734047.
149. Wu K, Yang L, Chen J, Zhao H, Wang J, Xu S, et al. miR-362-5p inhibits proliferation and migration of neuroblastoma cells by targeting phosphatidylinositol 3-kinase-C2beta. FEBS Lett. 2015;589(15):1911-9. doi: 10.1016/j.febslet.2015.05.056. PubMed PMID: 26073258.
150. Yang P, Ni F, Deng RQ, Qiang G, Zhao H, Yang MZ, et al. MiR-362-5p promotes the malignancy of chronic myelocytic leukaemia via down-regulation of GADD45alpha. Mol Cancer. 2015;14:190. doi: 10.1186/s12943-015-0465-3. PubMed PMID: 26545365; PubMed Central PMCID: PMCPMC4636774.
151. Tanoglu A, Balta AZ, Berber U, Ozdemir Y, Emirzeoglu L, Sayilir A, et al. MicroRNA expression profile in patients with stage II colorectal cancer: a Turkish referral center study. Asian Pac J Cancer Prev. 2015;16(5):1851-5. PubMed PMID: 25773836.
152. Ni F, Zhao H, Cui H, Wu Z, Chen L, Hu Z, et al. MicroRNA-362-5p promotes tumor growth and metastasis by targeting CYLD in hepatocellular carcinoma. Cancer Lett. 2015;356(2 Pt B):809-18. doi: 10.1016/j.canlet.2014.10.041. PubMed PMID: 25449782.
153. Xia JT, Chen LZ, Jian WH, Wang KB, Yang YZ, He WL, et al. MicroRNA-362 induces cell proliferation and apoptosis resistance in gastric cancer by activation of NF-kappaB signaling. J Transl Med. 2014;12:33. doi: 10.1186/1479-5876-12-33. PubMed PMID: 24495516; PubMed Central PMCID: PMCPMC3916099.
154. Zhai H, Song B, Xu X, Zhu W, Ju J. Inhibition of autophagy and tumor growth in colon cancer by miR-502. Oncogene. 2013;32(12):1570-9. doi: 10.1038/onc.2012.167. PubMed PMID: 22580605; PubMed Central PMCID: PMCPMC3422411.
155. Jin H, Yu M, Lin Y, Hou B, Wu Z, Li Z, et al. MiR-502-3P suppresses cell proliferation, migration, and invasion in hepatocellular carcinoma by targeting SET. Onco Targets Ther. 2016;9:3281-9. doi: 10.2147/OTT.S87183. PubMed PMID: 27330307; PubMed Central PMCID: PMCPMC4898420.
156. Sun LL, Wang J, Zhao ZJ, Liu N, Wang AL, Ren HY, et al. Suppressive role of miR-502-5p in breast cancer via downregulation of TRAF2. Oncol Rep. 2014;31(5):2085-92. doi: 10.3892/or.2014.3105. PubMed PMID: 24677135.

157. Larsen AC. Conjunctival malignant melanoma in Denmark: epidemiology, treatment and prognosis with special emphasis on tumorigenesis and genetic profile. Acta Ophthalmol. 2016;94 Thesis 1:1-27. doi: 10.1111/aos.13100. PubMed PMID: 27192168.
158. Ning MS, Kim AS, Prasad N, Levy SE, Zhang H, Andl T. Characterization of the Merkel Cell Carcinoma miRNome. J Skin Cancer. 2014;2014:289548. doi: 10.1155/2014/289548. PubMed PMID: 24627810; PubMed Central PMCID: PMCPMC3929981.
159. Wang W, Zhao LJ, Tan YX, Ren H, Qi ZT. Identification of deregulated miRNAs and their targets in hepatitis B virus-associated hepatocellular carcinoma. World J Gastroenterol. 2012;18(38):5442-53. doi: 10.3748/wjg.v18.i38.5442. PubMed PMID: 23082062; PubMed Central PMCID: PMCPMC3471114.
160. Wang W, Zhao LJ, Tan YX, Ren H, Qi ZT. MiR-138 induces cell cycle arrest by targeting cyclin D3 in hepatocellular carcinoma. Carcinogenesis. 2012;33(5):1113-20. doi: 10.1093/carcin/bgs113. PubMed PMID: 22362728; PubMed Central PMCID: PMCPMC3334515.
161. Xu G, Li JY. Differential expression of PDGFRB and EGFR in microvascular proliferation in glioblastoma. Tumour Biol. 2016;37(8):10577-86. doi: 10.1007/s13277-016-4968-3. PubMed PMID: 26857280.
162. Harvey H, Piskareva O, Creevey L, Alcock LC, Buckley PG, O'Sullivan MJ, et al. Modulation of chemotherapeutic drug resistance in neuroblastoma SK-N-AS cells by the neural apoptosis inhibitory protein and miR-520f. Int J Cancer. 2015;136(7):1579-88. doi: 10.1002/ijc.29144. PubMed PMID: 25137037.
163. Hong S, Bi M, Chen S, Zhao P, Li B, Sun D, et al. MicroRNA-520f suppresses growth of gastric carcinoma cells by target ATPase family AAA domain-containing protein 2 (ATAD2). Neoplasma. 2016;63(6):873-9. doi: 10.4149/neo_2016_606. PubMed PMID: 27565325.
164. Xiong Y, Zhang L, Holloway AK, Wu X, Su L, Kebebew E. MiR-886-3p regulates cell proliferation and migration, and is dysregulated in familial non-medullary thyroid cancer. PLoS One. 2011;6(10):e24717. doi: 10.1371/journal.pone.0024717. PubMed PMID: 21998631; PubMed Central PMCID: PMCPMC3187745.
165. Bi N, Cao J, Song Y, Shen J, Liu W, Fan J, et al. A microRNA signature predicts survival in early stage small-cell lung cancer treated with surgery and adjuvant chemotherapy. PLoS One. 2014;9(3):e91388. doi: 10.1371/journal.pone.0091388. PubMed PMID: 24637927; PubMed Central PMCID: PMCPMC3956664.
166. Liu C, Iqbal J, Teruya-Feldstein J, Shen Y, Dabrowska MJ, Dybkaer K, et al. MicroRNA expression profiling identifies molecular signatures associated with anaplastic large cell lymphoma. Blood. 2013;122(12):2083-92. doi: 10.1182/blood-2012-08-447375. PubMed PMID: 23801630; PubMed Central PMCID: PMCPMC3778551.
167. Gao W, Shen H, Liu L, Xu J, Xu J, Shu Y. MiR-21 overexpression in human primary squamous cell lung carcinoma is associated with poor patient prognosis. J Cancer Res Clin Oncol. 2011;137(4):557-66. doi: 10.1007/s00432-010-0918-4. PubMed PMID: 20508945.
